# Multi-omics profiling of high-carotenoid hybrid potato lines reveals coordinated metabolic reprogramming and associates with distinct tuber microbiota

**DOI:** 10.1038/s41538-026-00842-3

**Published:** 2026-07-04

**Authors:** Bo Zhang, Yiming Zhong, Blaise Pascal Muvunyi, Tingting Xu, Jiawei Liu, Xingyao Xiong, Xu Cheng

**Affiliations:** 1 Shenzhen Branch, Guangdong Laboratory of Lingnan Modern Agriculture, Key Laboratory of Synthetic Biology, Ministry of Agriculture and Rural Affairs, Agricultural Genomics Institute at Shenzhen, Chinese Academy of Agricultural Sciences, Shenzhen, China; 2 Yuelushan Laboratory, Changsha, China; 3 Hunan Provincial Engineering Research Center for Potatoes, College of Horticulture, Hunan Agricultural University, Changsha, China; 4 State Key Laboratory of Crop Stress Adaptation and Improvement, School of Life Sciences, Henan University, Kaifeng, China; 5 College of Agriculture, South China Agricultural University/National Engineering Research Center of Plant Space Breeding, Guangzhou, China; 6 Bama Yao Autonomous County Rural Revitalization Research Institute, Bama, China

**Keywords:** Biochemistry, Biotechnology, Microbiology, Plant sciences

## Abstract

Potato is a critical staple crop, and enhancing its carotenoid content is a promising strategy to improve its nutritional value. However, the synergistic mechanisms underlying carotenoid accumulation, superior nutritional traits, and the role of the endophytic microbiome remain unclear. Using an integrated multi-omics strategy, we systematically analyzed two high-zeaxanthin/lutein hybrids and four commercial cultivars. The hybrids accumulated significantly higher levels of zeaxanthin, lutein, and minerals, while exhibiting superior processing traits (e.g., higher dry matter/starch, lower reducing sugars). Integrated metabolomic and transcriptomic profiling revealed a coordinated upregulation of carotenoid and phenylpropanoid biosynthesis, alongside enrichment of stress-responsive phenolic acids. Notably, the endophytic microbiome in high-carotenoid tubers was distinct, dominated by Firmicutes and Proteobacteria, with genera like *Bacillus* and *Latilactobacillus* positively correlating with carotenoid content. Weighted gene co-expression network analysis identified a core regulatory module containing key genes (e.g., *CCD4*, *BCH2*) and novel transcription factors. Our findings elucidate a synergistic network linking metabolism, gene regulation, and the endophytic microbiome that collectively is associated with carotenoid accumulation and tuber quality. This provides critical targets for breeding nutritionally enhanced potatoes with desirable agronomic performance, supporting nutritional security and sustainable agriculture.

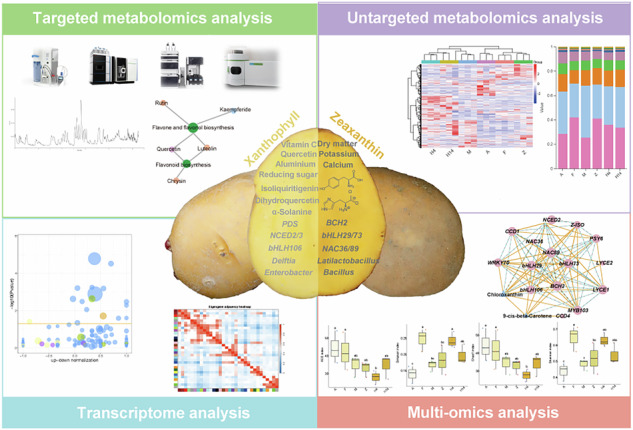

## Introduction

Potato (*Solanum tuberosum* L.) is the world’s most widely cultivated non‑cereal staple, producing over 300 million tons annually and underpinning global food security. Its edible tuber—a remarkable storage organ that originated from an ancient natural hybridization event—epitomizes the success of plant domestication^1^. This seminal work not only deciphers the hybrid origin of the tuber but also establishes the tuber as a classic model for studying organ innovation and specialized metabolism. As a dietary staple for nearly one billion people, it supplies complex carbohydrates, fiber, essential vitamins (C, B6) and minerals (potassium, magnesium), forming a solid nutritional foundation for human health^2–4^.

Beyond these established benefits, potatoes hold considerable and underexploited potential for nutritional enhancement. Yellow and orange‑flesh varieties are gaining consumer preference, reflecting the recognized antioxidant properties and vision‑health roles of carotenoids such as zeaxanthin and lutein^5,6^. These compounds scavenge reactive oxygen species, protect cellular integrity and help maintain metabolic homeostasis^7,8^. Natural variation among breeding lines offers a rich resource for targeted biofortification, presenting a sustainable route to address micronutrient gaps ^9^—an approach already validated in cereals such as maize^10^. However, this rich nutritional diversity remains a largely untapped resource in breeding programs, which have historically prioritized yield and processing qualities, often at the expense of comprehensive nutritional traits^11^.

Advances in plant science have revealed diverse routes to leverage this genetic potential. A number of established strategies are available to enhance carotenoid content in plants. These include metabolic engineering (e.g., *crtB* overexpression, RNAi fine-tuning)^5,12,13^ and transcriptional regulation mediated by specific transcription factors (e.g., from the MYB, BBX, NAC, MADS-box, and AP2/ERF families)^14,15^. Collectively, these approaches underscore the potential of exploiting natural allelic variation for crop improvement, offering promising alternatives to strategies that rely solely on transgenic methods.

An emerging paradigm in plant science recognizes that plant phenotypes, including metabolic profiles such as carotenoid accumulation, are shaped not only by the host genome but also by symbiotic endophytic microbes^16^. This view is supported by conceptual frameworks that highlight tight metabolome-microbiome coupling as a key determinant of plant traits, as evidenced in other plant systems where specific microbial communities are associated with secondary metabolite accumulation^17–19^. For example, in foxtail millet, a core microbiome coordinates enhanced seed yellowness and carotenoid content^16^, while in tea plants, the rhizosphere microbiome is associated with the synthesis of theanine, a key quality-related metabolite^18,20^. This positions the plant microbiome as a significant, yet underexplored, factor driving phenotypic variation.

Notably, the functional impact of these plant-derived compounds extends beyond the plant. Carotenoids mediate critical biological interactions in humans, influencing diet-microbiota-host dynamics by promoting beneficial gut taxa such as *Bifidobacterium* and *Lactobacillus* and facilitating microbial conversion of provitamin A to active retinol^21^, while their antioxidative properties help mitigate oxidative stress, bolster immunity, and lower risks of cardiometabolic and chronic diseases^22,23^. Thus, investigating the association between tuber carotenoids and the resident plant microbiome, and the nature of this association, represents a promising frontier for simultaneously optimizing crop traits and enhancing human nutritional and health outcomes.

Motivated by these opportunities, we employed a multi-omics framework to accelerate breeding of high-nutrition potato varieties. We analyzed two elite hybrids (H4 and H14)^24^ and four commercial cultivars, combining proximate composition, secondary metabolite profiling, mineral analysis, metabolic pathway mapping, transcriptomics and tuber microbiome characterization. By elucidating the interplay of metabolism, gene regulation and microbial communities, this study aims to provide actionable knowledge for unlocking the full nutritional potential of potatoes—advancing potato food science and supporting a sustainable future of nutritious, well-processed products.

## Results and discussion

### Comprehensive nutritional profiling reveals distinct metabolic strategies between hybrids and commercial cultivars

**Fig. 1:**
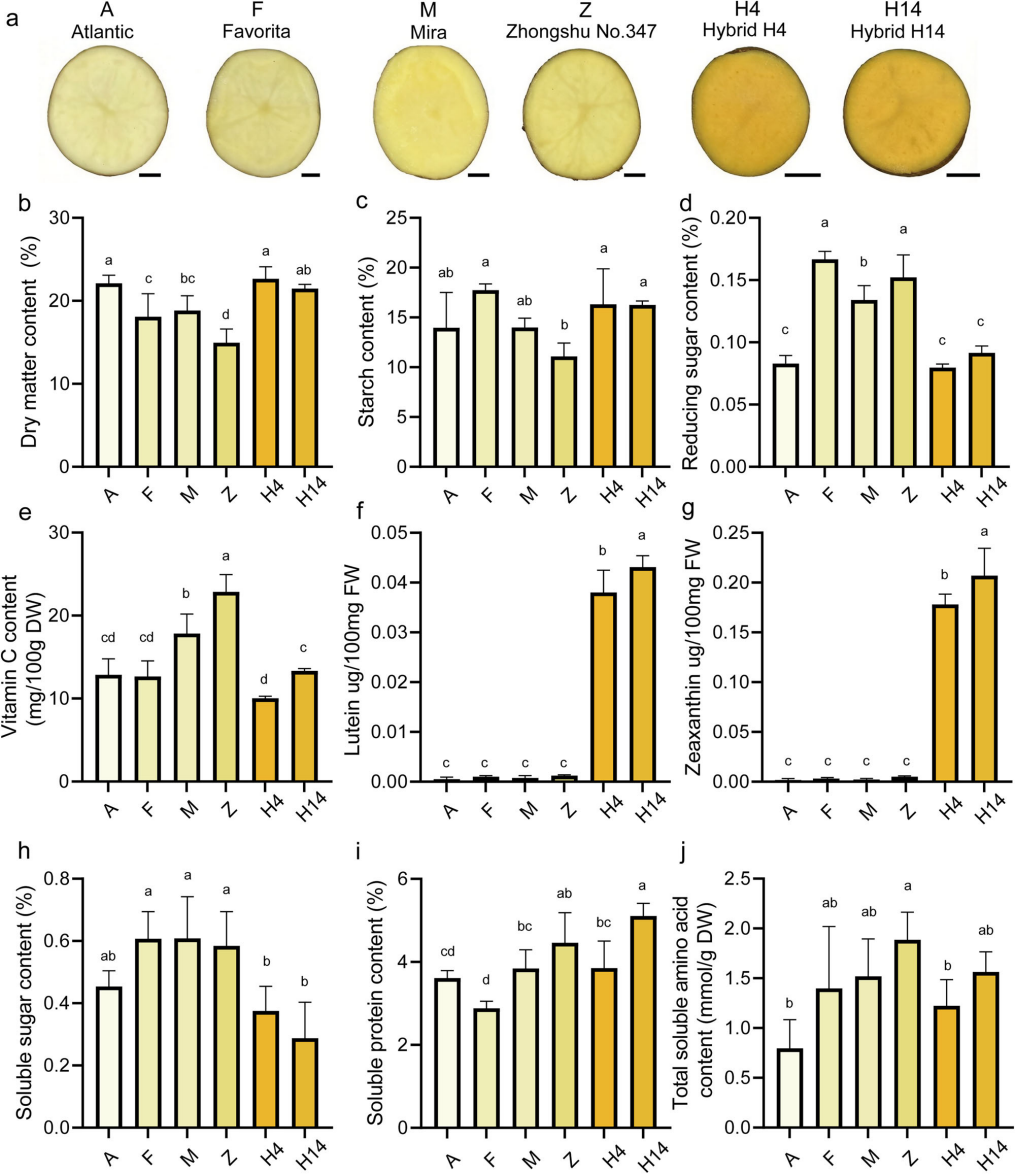
Phenotypic and nutritional characteristics of six potato samples. **a** Representative appearance of tuber flesh (A: Atlantic, F: Favorita, M: Mira, Z: Zhongshu No.347, H4: hybrid H4, H14: hybrid 22H14). Scale bar = 1 cm. Quantitative profiling of key nutritional and processing traits: dry matter (**b**), starch (**c**), reducing sugars (**d**), vitamin C (**e**), xanthophylls (**f**), zeaxanthin (**g**), soluble sugars (**h**), total soluble amino acids (**i**), and soluble protein (**j**). Bar colors correspond to flesh color of each cultivar. Different lowercase letters denote significant differences among cultivars (*P* < 0.05).

To elucidate the relationships between nutritional components, mineral nutrition and carotenoids, as well as the similarities and differences in carotenoid accumulation between hybrid varieties and commercial cultivars, we conducted a comparative analysis of the comprehensive nutritional profiles of six potato samples—four commercial cultivars (Atlantic [A], Favorita [F], Mira [M], Zhongshu No. 347 [Z]) and two elite hybrids (H4 and H14)^24^. The marked variation in flesh color—spanning from white (A) and light yellow (F, M) to yellow/dark yellow (Z) and vivid orange-yellow (H4, H14) (Fig. 1a, Supplementary Fig. 1)—prompted an integrated assessment of proximate composition, bioactive secondary metabolites, and essential mineral elements, revealing two divergent metabolic strategies linked to these pigmentation patterns and nutritional quality.

Among the six potato lines analyzed, carotenoid profiles and associated nutritional traits diverged markedly. H4 and H14 possessed intense orange-yellow flesh, reflecting the highest accumulation of zeaxanthin (0.17 ± 0.008 and 0.21 ± 0.02 µg/100 mg FW) and lutein (0.0431 ± 0.001 µg/100 mg FW, in H14) (Fig. 1f, g), pigments linked to antioxidant benefits and eye health, including protection against age‑related macular degeneration^6^. These hybrids also contained higher total amino acids (1.22 ± 0.21 and 1.56 ± 0.16 mmol/g DW) and soluble protein (5.10 ± 0.24% in H14, *P* < 0.05), indicating superior nutritional quality^25^. Notably, this enhanced nutritional profile was accompanied by favorable processing biochemistry, including high dry matter (22.65 ± 0.68% and 21.48 ± 0.40%) and starch content, coupled with low reducing sugars (0.08 ± 0.002% and 0.09 ± 0.004%; *P* < 0.05) (Fig. 1b–d) — traits that could potentially mitigate acrylamide formation during frying^26^, though direct processing validation is required. In contrast, Zhongshu No.347 (Z) was distinguished by the highest vitamin C level (22.86 ± 1.2 mg/100 g FW, *P* < 0.05; Fig. 1e), aligning with its potential for fresh consumption. Atlantic (A) served as the industrial benchmark with established processing utility^27^, while Favorita (F) and Mira (M) displayed moderate nutritional and processing characteristics. The co‑occurrence of high carotenoids, enhanced protein quality, and favorable processing traits in H4 and H14 positions them as premier genetic resources for breeding potatoes that intrinsically combine high nutritional value with robust agronomic performance.

Targeted metabolite profiling revealed a fundamental divergence in secondary metabolism (Supplementary Fig. 2, 3). The high-carotenoid hybrids (H4 and H14) accumulated significantly higher levels of stress-responsive phenolic acids (e.g., salicylic acid, vanillic acid, *trans*-ferulic acid), which can attenuate oxidative stress associated with carotenoid biosynthesis and are inferred to enhance tuber storage stability^28^, and may further potentially mitigate acrylamide formation during processing^29^. Conversely, commercial cultivars, particularly Atlantic (A) and Zhongshu No. 347 (Z), were enriched in specific flavonoids such as isorhamnetin, apigenin, and quercetin, which are known for antioxidant and membrane protective activities potentially contributing to postharvest stability^30,31^. This indicates that commercial cultivars primarily rely on the flavonoid pathway for pigmentation and antioxidant defense, whereas H4 and H14 utilize the carotenoid pathway, supplemented by a distinct phenolic acid profile.

Mineral profiling further delineated the hybrids, with H4 and H14 exhibiting superior accumulation of key minerals linked to carotenoid metabolism (Supplementary Fig. 4). They showed significantly elevated levels of potassium (K), associated with enhanced tuber quality and processing traits^13,32^, and calcium (Ca), which modulates carotenoid accumulation and suppresses catabolic genes^33^. Magnesium (Mg), an activator of key carotenoid biosynthetic enzymes^34^, was also enriched. The micronutrients iron (Fe) and zinc (Zn), critical for antioxidant capacity^4^, were elevated. Notably, positive correlations were found between total carotenoid content and the concentrations of Ca and Zn.

To evaluate the nutritional impact, a mineral contribution assessment was performed based on raw tuber mineral concentrations (Supplementary Table 1). The elite hybrids H4 and H14 exhibited outstanding profiles for key trace minerals. Specifically, a standard 250 g serving (fresh weight) of H14 could provide approximately 21% of the adult Recommended Dietary Allowance (RDA) for zinc. Both hybrids also served as excellent dietary sources of manganese (Mn), requiring the fewest servings to meet the RDA among all tested genotypes. Their contributions to copper (Cu) and iron (Fe) intakes were competitive with or superior to commercial cultivars^35^. Collectively, the integrated profiling reveals that the superior carotenoid accumulation in H4 and H14 is associated with a coordinated metabolic strategy favoring carotenoid and specific phenolic acid biosynthesis, coupled with enhanced accumulation of key minerals that may support its synthesis and stability^33^. These results, within the genetic scope of this study, position the examined high-carotenoid hybrids H4 and H14 as nutritionally enhanced materials, functioning as effective dietary sources, particularly for zinc and manganese.

Integrated nutritional profiling reveals distinct metabolic strategy tendencies between the two categories of potato varieties examined in this study. The high-carotenoid hybrids H4 and H14 exhibited a synergistic metabolic trait profile: on one hand, they possess a biochemical foundation of high dry matter, high starch, and low reducing sugar content, which suggests a potential for lower acrylamide formation during processing; on the other hand, they achieve pigmentation through the carotenoid biosynthetic pathway while co-accumulating specific stress-responsive phenolic acids. In contrast, the commercial cultivars used in this study (e.g., Atlantic and Zhongshu No. 347) displayed a pigmentation and antioxidant pattern more reliant on the flavonoid pathway. Furthermore, H4 and H14 demonstrated significantly superior accumulation of multiple key minerals (e.g., K, Ca, Mg, Zn, Fe), and the concentrations of these minerals showed significant positive correlations with carotenoid content, implying a potential synergistic role in supporting carotenoid synthesis and stability. In summary, within the limited genetic scope of this study, the high-carotenoid hybrids H4 and H14 present a composite profile that integrates potential superior processing quality, high nutritional value (notably as dietary sources of zinc and manganese), and a distinct secondary metabolic pattern, positioning them as valuable candidate materials for future potato breeding.

### Metabolomic profiling delineates distinct metabolic association networks in six potato samples

**Fig. 2:**
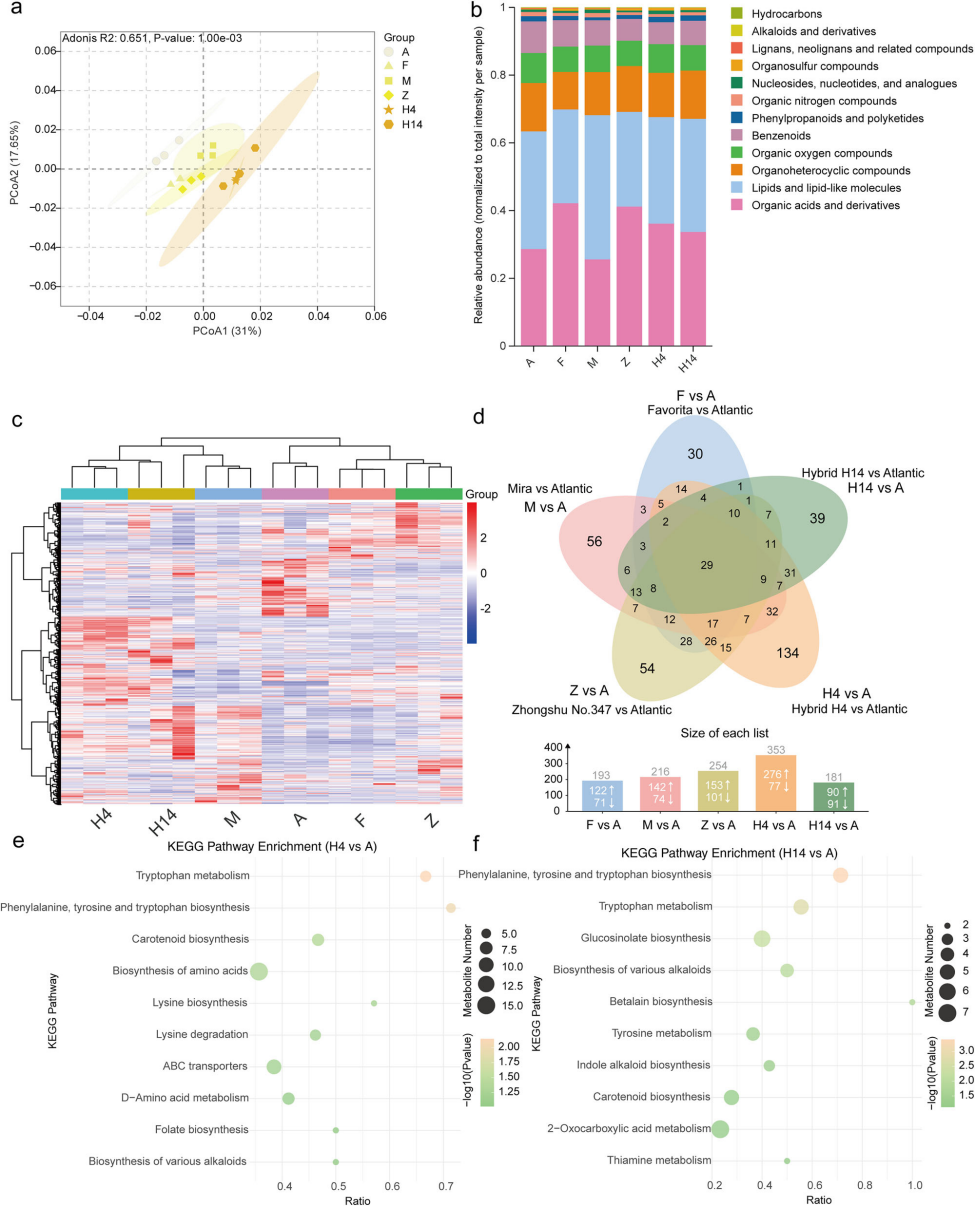
Comprehensive metabolomic analysis of six potato tuber samples. **a** PCoA illustrating the distribution patterns of samples. **b** Relative abundance of metabolite classes across groups. Y-axis: Relative abundance (normalized to total intensity per sample); X-axis: Groups (A, F, M, Z, H4, H14). Colors represent different metabolite classes (legend). **c** Heatmap of metabolite abundance. Rows: Metabolites; Columns: Groups (A, F, M, Z, H4, H14). Color scale: Z-score of metabolite abundance (red = higher than mean, blue = lower than mean; scale range: -2 to 2). Hierarchical clustering (top: metabolites; left: groups) shows similarity patterns. **d** Venn diagram of differentially abundant metabolites across group comparisons. Comparisons: F vs A (Favorita vs Atlantic), M vs A (Mira vs Atlantic), Z vs A (Zhongshu No.347 vs Atlantic), H4 vs A (Hybrid H4 vs Atlantic), H14 vs A (Hybrid H14 vs Atlantic). Numbers in each region: Count of unique/overlapping metabolites. Bar plot below: Size of each metabolite list (upregulated ↑; downregulated ↓). **e** Top KEGG pathways enriched in ‘H4 vs A’. **f** Top KEGG pathways enriched in ‘H14 vs A’. e and f Bubble size: Number of differentially abundant metabolites in the pathway; Bubble color: -log₁₀(P-value) (darker color = more significant); X-axis: Ratio (number of differential metabolites / total metabolites in pathway); Y-axis: KEGG pathway. Pathways are sorted by -log₁₀(P-value) in descending order (only significant pathways, *P* < 0.05, are shown).

To further decipher the metabolic basis underlying the phenotypic differences, we performed widely targeted metabolomic profiling on tuber samples from the six potato genotypes using UPLC–MS/MS. Principal coordinate analysis (PCoA) revealed clear clustering of the samples according to their genetic background and phenotype (Fig. 2a). The two high-carotenoid hybrids, H4 and H14, formed a distinct, tight cluster, clearly separated from the commercial cultivars. Among the commercial lines, Atlantic (A) was an outlier, while Favorita (F), Mira (M), and Zhongshu No. 347 (Z) grouped together. This clustering pattern is strongly associated with flesh color and carotenoid content (Fig. 1), indicating that the overall metabolic profile covaries with these key traits.

Across 12 major metabolite classes, 886 compounds were identified (Fig. 2b, Supplementary Data 1), with organic acids, lipids, nitrogen heterocycles and oxygen‑rich organics accounting for >90% of detections^36^. Hierarchical clustering (Fig. 2c) confirmed that H4 and H14 shared highly similar metabolic modules, reflecting conserved regulatory mechanisms, whereas A showed marked upregulation of flavonoids and phenolic acids, and F and Z exhibited metabolic patterns comparable to A, a pattern consistent with the targeted analyses shown in Supplementary Fig. 2.

**Fig. 3:**
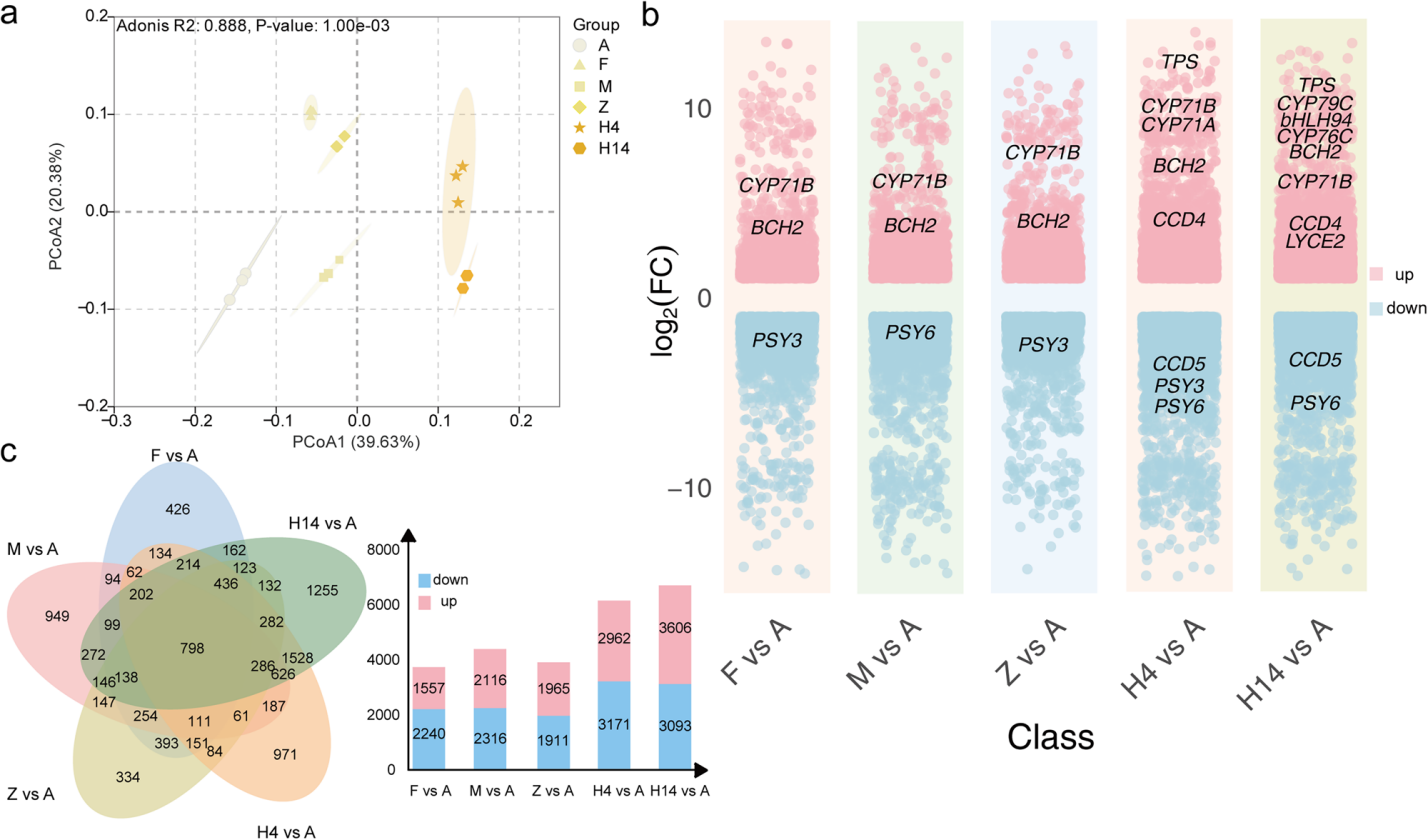
Comprehensive transcriptomic analysis of six potato tuber samples. **a** Principal coordinates analysis illustrating the clustering pattern of tuber transcriptomes. **b** Volcano plots of differentially expressed genes (DEGs) identified in five pairwise comparisons (H4 vs. A, H14 vs. A, F vs. A, M vs. A, and Z vs. A). Genes meeting the thresholds of adjusted *P* < 0.05 and |log₂(fold change)| ≥ 1 are highlighted in pink (upregulated) and blue (downregulated). Key carotenoid pathway genes are labeled. **c** Venn diagram depicting the overlap of DEG sets (both up- and downregulated) identified across the five comparisons. The bar chart below quantifies the total number of DEGs in each comparison.

Pairwise comparison against A revealed distinct reprogramming: H4 vs. A displayed the largest shift, with 353 differentially abundant metabolites (276 upregulated, 77 downregulated; Fig. 2d), signifying extensive metabolic reconfiguration. By contrast, F, M and Z consistently upregulated flavonoids and phenolic acids relative to A—compounds implicated in flesh coloration via antioxidant activity and pigment stabilization^37^ (Supplementary Fig. 5). All non‑A samples (H4, H14, F, M, Z) also showed a coordinated shift toward higher amino acid levels (e.g., tyrosine, tryptophan) and reduced solanine (Supplementary Fig. 6a), suggesting a potential improvement in nutritional quality and diminished defense compound accumulation.

Within F, M and Z, key pathway changes included enrichment of flavonoid biosynthesis and phenolic acid accumulation, consistent with a metabolic strategy that could support coloration through antioxidant stabilization and oxidative stress response^28,30^. In H4 and H14, differential metabolites mapped strongly to the carotenoid biosynthesis pathway (ko00906; Fig. 2e, f), consistent with their elevated carotenoid levels (Fig. 1f, g). Reprogramming encompassed multiple coordinated pathways: activation of the shikimate pathway was associated with increased phenylalanine biosynthesis (Supplementary Fig. 6a)—a precursor for flavonoids and lignin^28^ —which raises the possibility that intermediates such as 4‑coumaroyl‑CoA could contribute to carotenoid synthesis via shared isopentenyl pyrophosphate (IPP) pools^12,38^; and upregulation of arginine and proline metabolism, linked to osmotic balance and stress tolerance^39^. These metabolite changes are associated with functions that may confer multifunctional benefits—such as scavenging free radicals, mitigating oxidative degradation, and potentially supplying precursors for carotenoids and other quality-related compounds.

K‑means clustering (Supplementary Fig. 6b, Supplementary Data 1) grouped sesquiterpenoids and carotenoids in subclass 4, suggesting a potential co-regulation mediated by shared IPP pools. This association hints at the possibility of competitive IPP utilization or feedback mechanisms^12^, a hypothesis that warrants future validation, particularly in high-carotenoid lines such as H4 and H14. Importantly, these interpretations are based on steady-state metabolite data; direct evidence of carbon flux reallocation among these pathways would require future flux analysis. Beyond terpenoids, the analysis identified metabolites associated with processing quality that may aid carotenoid retention during storage and thermal processing^4^. Sugar alcohols (e.g., sorbitol) could help maintain cellular hydration to limit dehydration‑induced oxidative stress^40^. Hydrophobic amino acids (e.g., leucine) may stabilize membranes; thiamine provides antioxidant protection^41^. and polyunsaturated fatty acids contribute to membrane integrity^42^. Collectively, the observed enrichment of these compounds supports a model in which they may act synergistically to protect carotenoids from oxidative damage.

Taken together, our metabolomic network analysis of the six genotypes studied here suggests that the high-carotenoid hybrids H4 and H14 may achieve their trait advantages through a coordinated metabolic module. Beyond the core enhancement of carotenoid biosynthesis, concomitant changes in specific amino acids, sugar alcohols, membrane lipids, and vitamins likely constitute a protective metabolic network that mitigates oxidative damage and stabilizes the cellular environment. This network could metabolically underpin their high carotenoid content and contribute to its stability. These findings, based on steady-state metabolite associations within the examined set of genotypes, provide a foundational metabolic framework. They generate testable hypotheses for future research aimed at dissecting the molecular mechanisms of carbon partitioning and flux regulation, and at determining whether the observed metabolic coordination is a general feature of high-carotenoid potatoes or specific to certain genetic backgrounds.

### Transcriptome profiling uncovered nutrient and processing-related genes in six potato samples

PCoA of gene expression profiles clearly separated the six potato samples into two clusters along PCoA1: a low‑carotenoid group (cultivars A, F, M, Z) and a high‑carotenoid group (hybrids H4 and H14) (Fig. 3a), mirroring the metabolomic clustering pattern (Fig. 2a). This transcriptional divergence clearly separates the high-carotenoid hybrids from the commercial cultivars, underscoring that the genetic programs for high carotenoid accumulation in H4 and H14 involve extensive transcriptomic rewiring compared to standard varieties.

Pairwise comparisons against the commercial cultivar A revealed that the transcriptional shifts were far more extensive in the two high-carotenoid hybrids, with 6133 and 6699 DEGs identified in H4 and H14, respectively (Fig. 3b, Supplementary Data 2, 3). In contrast, the shifts in the other commercial cultivars (F, M, and Z) were more moderate, ranging from 3797 to 4432 DEGs (Fig. 3b, Supplementary Data 4–6). Notably, H4 and H14 themselves were transcriptionally highly similar, sharing 92.9% of their DEGs and differing by only 566 genes (Fig. 3c). This pattern underscores a highly conserved transcriptional network specific to the high-carotenoid hybrid group, which is distinct from the more varied transcriptional landscapes observed among commercial cultivars.

Gene abbreviations: *BCH2*, Beta-carotene hydroxylase 2; *bHLH94*, basic helix-loop-helix transcription factor 94; *CCD4/CCD5*, carotenoid cleavage dioxygenase 4/5; *CYP71A/CYP71B/CYP76C/CYP79C*, cytochrome P450 family 71 subfamily A, family 71 subfamily B, family 76 subfamily C, family 79 subfamily C; *LYCE2*, lycopene epsilon-cyclase 2; *PSY3/PSY6*, phytoene synthase 3/6; *TPS*, terpenoid cyclase/protein prenyltransferase superfamily.

**Fig. 4:**
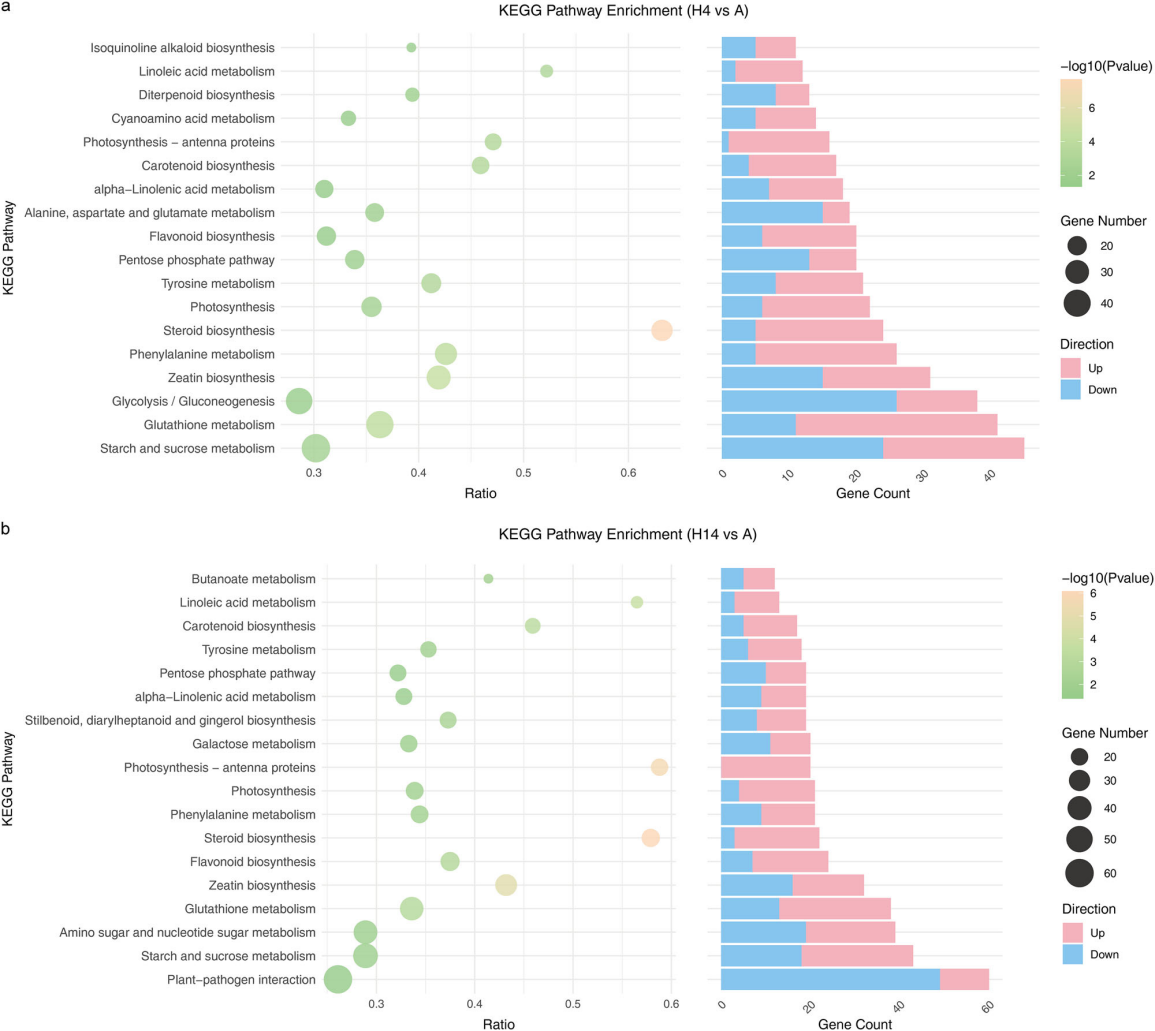
KEGG pathway enrichment analysis of DEGs in the comparisons of H4 vs. A and H14 vs. A. **a** H4 vs. A; **b** H14 vs. A. In each panel, the left scatter plot displays significantly enriched KEGG pathways ranked by the number of mapped DEGs (dot size). The x-axis indicates the “Ratio” (number of DEGs in the pathway / total number of pathway genes in the reference genome), and the y-axis lists pathway names. Dot color intensity represents the enrichment significance [-log₁₀(adjusted *P*-value)]. The adjacent bar chart provides a detailed breakdown of the transcriptional regulation direction for each enriched pathway, with bars quantifying the number of upregulated (pink) and downregulated (blue) DEGs.

Key KEGG pathways co-upregulated in the hybrid group (H4 and H14) compared to all commercial cultivars included carotenoid biosynthesis (ko00906), phenylalanine metabolism (ko00360), and starch-sucrose metabolism (ko00500) (Fig. 4a, b). The co-upregulation of these pathways suggests a model of integrated metabolic control that may contribute significantly to carotenoid accumulation. Carotenoid biosynthesis pathway genes were coordinately upregulated with genes in the tricarboxylic acid (TCA) cycle, the pentose phosphate pathway (providing NADPH)^43^, and phytohormone signaling pathways (ABA-mediated stress responses and auxin-regulated growth)^5^. This transcriptional coordination implies potential metabolic crosstalk, where the TCA cycle could potentially supply precursors for isopentenyl pyrophosphate (IPP) biosynthesis^44^, supporting enhanced carotenoid production under uniform growth conditions.

Phenylalanine metabolism was markedly enriched in H4 and H14. Its co-upregulation with carotenoid biosynthesis suggests a potential coordinated strategy, which could balance structural integrity (via lignin)^12^ and antioxidant capacity (via carotenoids and flavonoids)^31^. The upregulation of the shikimate pathway indicates an enhanced capacity to produce aromatic amino acids. These amino acids could, in turn, support flavonoid synthesis for oxidative defense, lignin deposition for mechanical strength, and provide phenylpropanoid precursors that may stabilize carotenoids during storage^45^.

The upregulation of starch–sucrose metabolism in H4 and H14 is consistent with a model in which carbon could be channeled toward carotenogenesis, potentially by providing glyceraldehyde‑3‑phosphate and erythrose‑4‑phosphate for the MEP and MVA pathways^12^. This transcriptional reprogramming aligns with not only elevated carotenoid levels but also traits beneficial for industrial use, such as increased starch content linked to improved tuber shelf life and processing stability^46^. By contrast, low‑carotenoid cultivars (F, M, Z) specifically enriched flavonoid biosynthesis (Supplementary Fig. 7), indicating their reliance on alternative, flavonoid-based stress‑adaptation strategies.

Collectively, the observed co-upregulation of phenylalanine metabolism, starch–sucrose partitioning and carotenoid biosynthesis pathways highlights a tractable, multi-pathway transcriptional module. Targeting the coordinated regulation of these linked pathways presents a promising strategy for breeding potato varieties with significantly enhanced carotenoid content alongside improved nutritional quality.

### Combined metabolomic and transcriptomic analysis uncovers a coordinated regulatory network for carotenoid accumulation

**Fig. 5:**
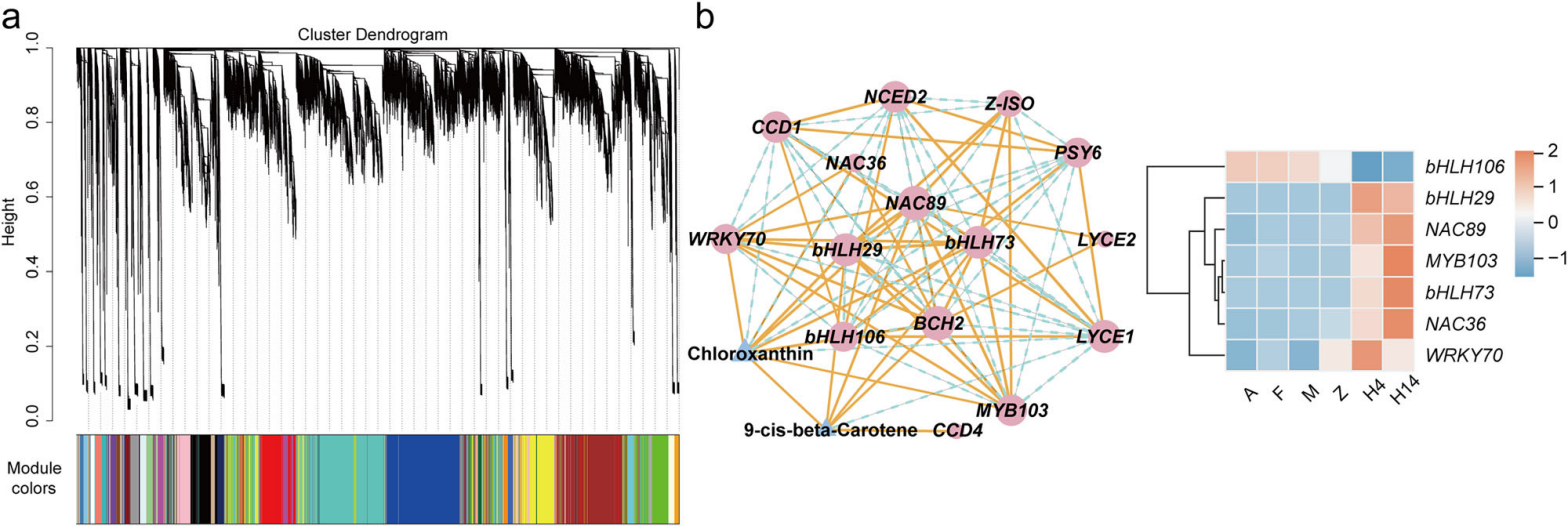
Weighted gene co‑expression network analysis and transcriptional network of carotenoid biosynthesis. **a** Hierarchical clustering dendrogram of 33 gene modules identified by WGCNA. The color bar (right) indicates module assignment. The turquoise module, which is strongly correlated with carotenoid accumulation, is highlighted. **b** Regulatory network derived from the key carotenoid-associated module. Pink nodes represent genes, and blue triangles represent metabolites. Orange solid edges denote significant positive correlations, and blue dashed edges denote significant negative correlations. Key genes in the carotenoid pathway are labeled, including *PSY6* (phytoene synthase 6), *LYCE1/2* (lycopene epsilon‑cyclase 1/2), and *CCD1/4* (carotenoid cleavage dioxygenase 1/4). The putative regulatory functions of core transcription factors are indicated, including *bHLH106* (a negative regulator), *bHLH29/73* (positive regulators enhancing *LYCE* and repressing *CCDs*), *MYB103* (positive regulator of *PSY*), *NAC36/89* (positive regulators linking carotenoid metabolism to development/stress), and *WRKY70* (a positive stress-responsive regulator).

Integration of targeted and widely targeted metabolomics with weighted gene co-expression network analysis (WGCNA) identified a regulatory network encompassing 33 co-expressed modules (Fig. 5a, b; Supplementary Fig. 8a, b; Supplementary Data 7). The turquoise module was strongly enriched in key carotenoid biosynthesis genes, including phytoene synthase (*PSY6*), phytoene isomerase (*Z-ISO*), lycopene ε-cyclase (*LYCE1/LYCE2*), β-carotene hydroxylase (*BCH2*), carotenoid cleavage dioxygenases (*CCD1/CCD4*), and abscisic acid synthase (*NCED2*) (Supplementary Fig. 8c, Supplementary Data 8). These genes showed robust correlations (*r* > 0.60) with carotenoid levels, and RT-qPCR confirmed *BCH2* as a positive regulator driving metabolic divergence (Supplementary Data 8, Supplementary Fig. 9). Transcriptomic and RT-qPCR data were highly consistent across nine core genes in all six samples (Supplementary Fig. 9), confirming technical reliability.

Previous studies link a large inversion variant in the *BCH2* promoter to elevated expression and enhanced flesh yellowness in yellow-fleshed tubers^47^. Functional analysis indicated that *PSY* acts as the rate-limiting enzyme for lycopene synthesis, *LYCE1/LYCE2* divert ζ-carotene toward branched-chain carotenoids, *CCD1/CCD4* modulate cleavage equilibrium, and *NCED2*-mediated ABA biosynthesis provides feedback regulation^5,12,33^. K-means clustering defined Cluster 2, in which gene upregulation synchronized with carotenoid accumulation (Supplementary Fig. 8b) and was enriched for *BCH2*, *PSY6* and *Z-ISO*, validating the functional specificity of the turquoise module.

Beyond structural genes, seven novel transcription factors (TFs) from the MYB, bHLH, NAC and WRKY families were identified as tuber carotenoid regulators (Fig. 5b). WRKY binds to W-box motifs in target promoters, NAC activates *PDS2/4* promoters, and MYB/bHLH fine-tune metabolic flux via differential regulation of *PSY* and *LCYb*^15,31,34^. Promoter structural variation in *BCH2* and ABA signaling act synergistically to shape cultivar-specific pigment patterns^48^.

**Fig. 6:**
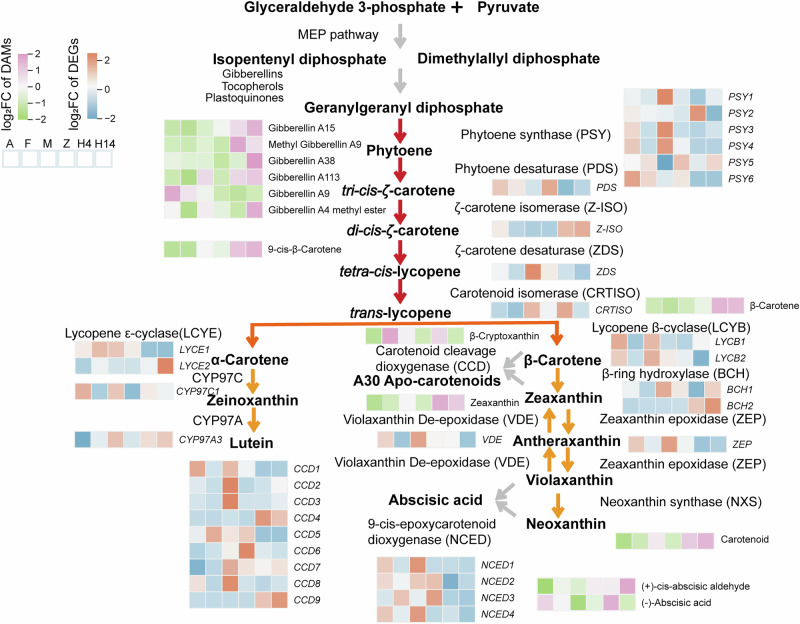
Summary of carotenoid biosynthesis, transcriptional regulation, and differential expression. Schematic of the carotenoid biosynthesis pathway, from MEP-derived precursors (glyceraldehyde-3-phosphate and pyruvate) to apocarotenoids. Red arrows denote upstream steps to *trans*-lycopene; orange arrows denote branch points initiating cyclic carotenoid synthesis via lycopene ε- or β-cyclation; gray arrows denote key partial pathways. Heatmaps display the expression profiles of DEGs (left panel; orange-blue color scale) and abundances of differentially expressed metabolites (DEMs, right panel; purple color scale) across the six genotypes (A, F, M, Z, H4, H14). Color intensity represents the log₂-transformed fold change relative to the respective mean, as indicated by the adjacent color bars. Abbreviations are defined in the main text.

This integrated analysis delineates a three-dimensional regulatory framework (Fig. 6): (1) Metabolic pathway coordination — *PSY6*/*Z-ISO* as rate-limiting steps, *LYCE1/LYCE2* as modifiers, *CCD1/CCD4* as catabolic nodes, and *NCED2* for hormonal feedback; (2) Transcriptional stratification – WRKY– NAC networks interacting with promoter variation; (3) Phenotypic plasticity emerging from genotype–environment interactions. The turquoise module unites these layers, illustrating a model in which transcriptional regulation and genetic variation act in concert to underpin cultivar-specific carotenoid accumulation in potato tubers, thereby informing precision breeding for nutritionally enhanced and visually appealing food crops.

### Microbiome composition in potato tuber flesh: structure, diversity, and functional correlations

**Fig. 7:**
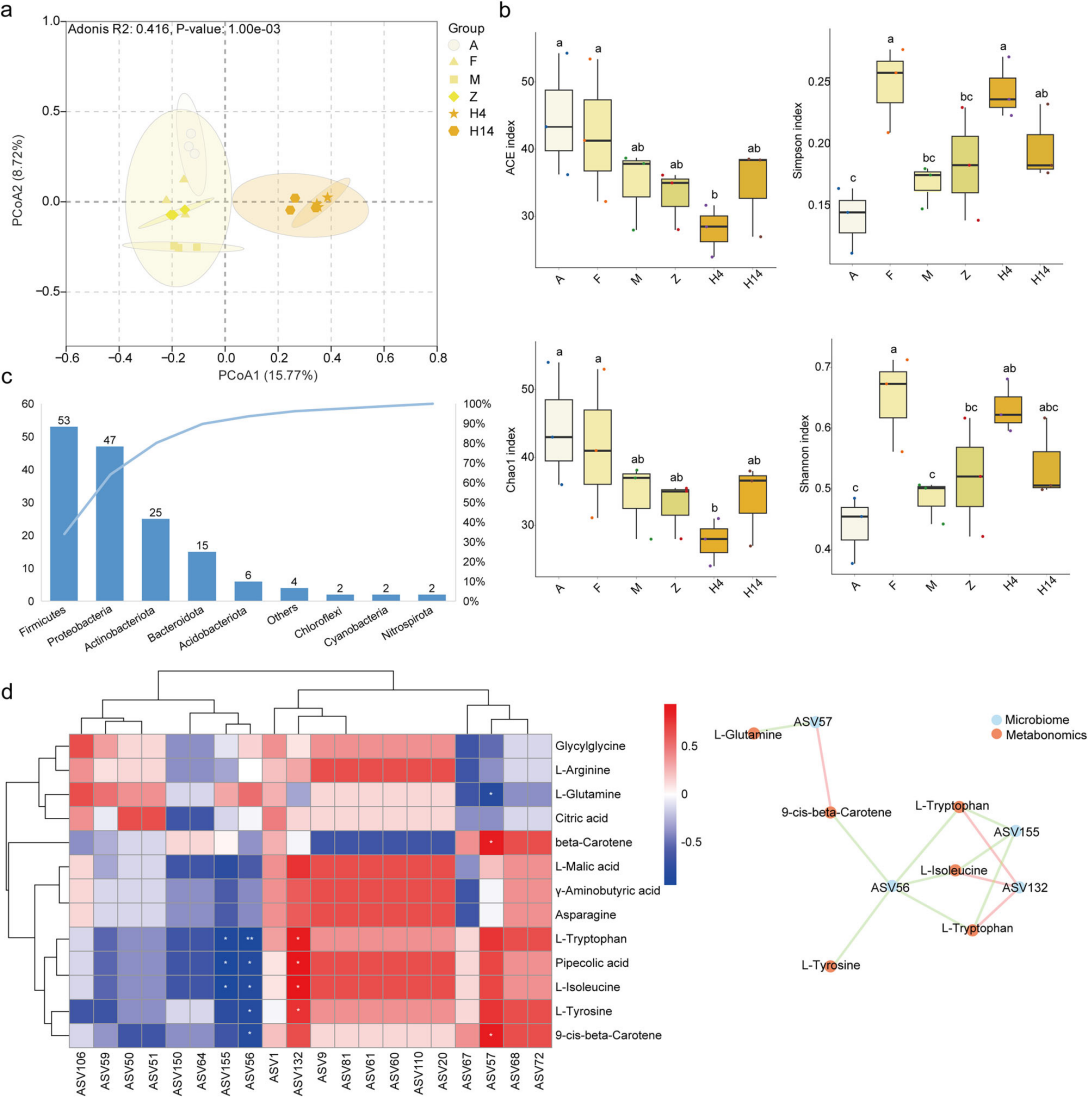
Comprehensive microbiome analysis of six potato tuber flesh samples. **a** PCoA based on Bray-Curtis dissimilarity, illustrating microbial community structure and sample clustering patterns. **b** Bar charts comparing α-diversity indices, including species richness (Chao1, ACE) and diversity (Shannon, Simpson). Statistical differences among groups were assessed by one-way ANOVA followed by Tukey’s post-hoc test (*P* < 0.05). **c** Phylum-level taxonomic composition of the potato tuber flesh microbiome. **d** Integrated correlation network analysis between differentially abundant ASVs and metabolites. (Left) Spearman correlation heatmap (ASVs = rows, metabolites = columns); significant correlations are indicated by *P < 0.05 and **P < 0.01. (Right) Network topology with ASVs (blue nodes), metabolites (pink nodes), and edges representing correlation strength and direction (red = positive, green = negative).

Potato tuber flesh, a hypoxic and sugar‑rich storage tissue, constitutes a unique yet underexplored microbial niche. Here, we systematically profiled bacterial community structure across six representative tuber flesh samples using 16S rRNA gene amplicon sequencing, to elucidate potential microbiome–tuber interactions (Supplementary Table 2). PCoA (PCoA1 explaining 15.77% of variance) together with Adonis testing (R² = 0.416, *P* <  0.05) resolved samples into two distinct clusters: cultivars A, F, M, Z and high‑carotenoid hybrids H4, H14 (Fig. 7a). Alpha‑diversity indices (ACE, Chao1, Simpson, Shannon) further revealed significant cultivar‑specific differences (Fig. 7b), indicating that microbial assembly is associated with genetic background and carotenoid accumulation phenotype.

Taxonomic profiling identified Firmicutes and Proteobacteria as dominant phyla (Fig. 7c), both previously implicated in secondary metabolite biosynthesis, microbial fermentation and plant tissue stress responses^49^. Co-abundance genus clustering (K-means) delineated two groups significantly enriched in H4 and H14 and positively correlated with carotenoid content (Supplementary Data 9, Supplementary Fig. 10). These clusters were dominated by *Bacillus* and *Latilactobacillus*, genera that have been reported in other systems in contexts related to carotenoid stability and pigment metabolism. For instance, *Bacillus* can potentially enhance carotenoid stability by lowering local pH via organic acid production^50^—a natural, additive-free strategy relevant to food preservation and extended shelf life. Metabolic engineering studies further demonstrate that *Bacillus* strains overexpressing farnesyl diphosphate synthase (*FPPS*) while disrupting competing branch-pathway genes (e.g., *yisP*) markedly increase C30 carotenoid yields^51^, suggesting a possible metabolic potential that could be relevant in the tuber context. *Latilactobacillus* may be involved in potentially analogous pigment-metabolism mechanisms, as inferred from bridging plant and human microbiome research^52^. It is important to note that these interpretations are based on associations and knowledge from other systems; causal roles for these microbial genera in potato tuber carotenoid metabolism remain to be experimentally validated.

Microbiome–metabolite interaction network analysis (Fig. 7d) uncovered ASV-level associations critical for food quality and safety: ASV57 (*Bacillus*) abundance positively correlated with 9-*cis*-β-carotene (*r* = 0.88, *P* = 0.02) and β-carotene (*r* = 0.65, *P* = 0.012), but negatively with L-glutamine (*r* = −0.81, *P* = 0.045), suggesting a potential link to amino acid metabolic regulation alongside carotenoid accumulation. ASV132 (*Paucibacter*) showed strong positive correlations with proline, tyrosine and tryptophan (*P* < 0.05), whereas ASV56 (*Bacillus*) and ASV155 exhibited significant negative correlations (*P* < 0.05) (Supplementary Data 10), highlighting potential microbial associations with precursor pools for both carotenoids and phenolic compounds. When discussing such microbiome-metabolome linkages, it is prudent to frame them within an associative rather than a causal framework, as they may reflect shared responses to the plant metabolic environment or complex multi-trophic interactions^53^.

Our integrated multi-omics analysis reveals that the superior nutritional quality of elite carotenoid-biofortified potato hybrids (H4 and H14) is underpinned by a coordinated, multi-layered network. This network is characterized by: (1) transcriptional reprogramming of core metabolic pathways (carotenoid, phenylalanine, starch-sucrose), with elevated expression of key genes such as *BCH2*; (2) an associated metabolic profile rich in provitamin A carotenoids, protective phenolic acids, and essential minerals; and (3) a distinct tuber microbiome assembly, where specific bacterial genera (e.g., *Bacillus*, *Latilactobacillus*) correlate with carotenoid content. In contrast, the commercial cultivars examined here primarily employ flavonoid-based metabolic strategies. Collectively, our findings demonstrate that the enhanced nutritional phenotype arises from extensive host genetic and metabolic reprogramming, which correlates with a specific microbiome profile. While these patterns are well-characterized in the studied hybrids, we note a limitation: only two elite hybrids represent the high-carotenoid phenotype. Future work involving a broader set of genetic materials is needed to determine the generalizability of these observed patterns and to delineate whether they are carotenoid-specific, hybrid-specific, or dependent on a particular genetic background. Furthermore, the documented microbial associations, while robust, are correlative; their causal, contributory, or responsive role in carotenoid traits remains a key question for future functional studies.

These mechanistic insights take on profound significance when contextualized within the evolutionary framework of the tuber itself. The recent elucidation of the potato tuber’s hybrid origin addresses the fundamental “why” of this organ’s existence^1^. We address the pivotal question of ‘how’—namely, how to enhance the nutritional output of this evolutionarily shaped organ. We demonstrate that such optimization is achieved via the precise modulation of internal regulatory networks—including transcriptional reprogramming, coordinated metabolic modules, and microbiome-associated signatures—that collectively enhance the accumulation of valuable compounds. Consequently, a coherent framework emerges: the tuber’s hybrid origin establishes an evolutionary foundation, while human selection—exemplified by modern breeding—harnesses a molecular toolkit to reprogram its internal regulatory networks. Future breeding strategies can integrate the deep genetic diversity revealed by evolutionary studies with the precise regulatory targets defined here to design the next generation of nutrient-dense potato varieties. This systems-level understanding provides a concrete set of biomarkers and breeding targets for developing nutritionally dense potato varieties with elevated carotenoid profiles and improved health-promoting metabolites.

## Methods

### Plant materials and sample processing

Four commercial potato cultivars (Atlantic [A], Favorita [F], Mira [M], Zhongshu No.347 [Z]) and two elite hybrids (H4 and H14) were grown under controlled field conditions at the Agricultural Genomics Institute Shenzhen Experimental Station (China). H4 and H14 were developed via a systematic breeding pipeline: highly homozygous diploid inbred lines ( >97% genome-wide homozygosity) were generated through multi-generational selfing combined with genomic selection; parental lines with low genomic variant overlap ( <17%) were crossed to maximize heterosis. After evaluation under standard agronomic practices, H4 and H14 were selected for superior nutritional quality^24^. All genotypes received NPK fertilizer (120‑60‑80 kg ha⁻¹) and were harvested at commercial maturity (120 days after planting; Supplementary Fig. 1).

Tubers were transported to the laboratory within 2 h post-harvest and standardized as follows: (i) surface decontamination with deionized water; (ii) equilibration at 4 °C (85% relative humidity) for 24 h. For multi‑omics assays, uniform tubers (10–15 per biological replicate, three independent replicates) were aseptically peeled, flash-frozen in liquid nitrogen, and stored at −80 °C (flesh tissue only). All procedures followed RNase‑free protocols, using analytical‑grade reagents (Sigma‑Aldrich) and HPLC‑grade solvents (Fisher Scientific).

### Proximate composition analysis

Total solids were determined gravimetrically: samples were dried at 105 ± 1 °C for 72 h until constant weight (three measurements per replicate). Starch was extracted by sequential sonication (50 Hz, 5 min), centrifugation, ethanol precipitation (95% [v/v], pH 6.0), and lyophilization ( − 80 °C, 48 h). Total soluble sugars were quantified by the anthrone–sulfuric acid method; reducing sugars were measured by dinitrosalicylic acid (DNS) assay with D‑glucose calibration^46^. VitaminC (Vc) was analyzed by LC–MS/MS (Acquity CSH C18, 2.1 × 150 mm, 1.7 μm; 0.1% formic acid/water–methanol, 0.3 mL min⁻¹; MRM m/z 175 → 115). Tuber samples (0.5 g) were homogenized (TissueLyser II, 30 Hz, 2 min) in 5 mL extraction buffer (50 mM EDTA, 1% H₃PO₄, 5 mM TCEP, pH3.0), centrifuged (12,000 × *g*, 15 min, 4 °C). Soluble protein was determined by Bradford assay (BSA standard)^54^. Free amino acids were quantified with a commercial kit (Boxbio) per manufacturer’s instructions.

### Carotenoid extraction and analysis

Carotenoids were extracted and saponified as described^55^ with modifications. Briefly, 100 mg tuber tissue was pre‑extracted with ethanol, saponified with KOH, washed with hexane, dried, and reconstituted in methanol for HPLC analysis.

HPLC analysis followed published procedures^56^, using a UHPLC-DAD system (Thermo DGLC) equipped with a YMC Carotenoid S‑3 column (3 μm, 4.6 × 150 mm; 40 ± 0.5 °C). Gradient elution (methanol/methanol‑methyl tert‑butyl ether‑water) was performed at 1.00 ± 0.02 mL min⁻¹, detection at 450 nm (slit 4 nm), injection volume 2 μL (autosampler at 4 ± 0.5 °C). Quality control samples were injected every 10 runs in randomized order. Zeaxanthin (CAS: 144‑68‑3) and lutein (CAS: 127‑40‑2; Sigma‑Aldrich) standards were used to construct external calibration curves for quantification.

### Phenolic acid and flavonoid extraction and analysis

Sample preparation followed a reported method^57^ with modifications. Tuber flesh (50 mg) was mixed with 0.5 mL extraction solution and 0.2 mL 50% (v/v) HPLC‑grade acetonitrile containing 2‑chloro‑L‑phenylalanine (internal standard) for UHPLC–MS/MS analysis. Separation was carried out on a Waters HSS T3 column (50 × 2.1 mm, 1.8 μm) with mobile phases A (ultrapure water) and B (acetonitrile), both containing 0.1% formic acid. Flow rate: 0.3 mL min⁻¹; column temperature: 40 °C; injection volume: 2 μL. Gradient: 0–2 min, 90% A; 6–9 min, 40% A; 9.1–12 min, 90% A. MS was operated in negative ion mode (m/z 100–900; full scan with data‑dependent MS²). Optimized parameters: capillary voltage 3.0 kV; sheath/auxiliary/sweep gas flows 40/15/1 (arb. units); ion transfer tube 320 °C; vaporizer 350 °C.

### Mineral element quantification

For digestion, 0.1 g of finely ground tuber flesh was placed in a microwave digestion vessel, followed by 5 mL concentrated nitric acid. Digestion was performed using a pre‑optimized microwave program^58^.

Elemental analysis was conducted on a Thermo Fisher iCAP TQ ICP‑MS system (USA) in He/O₂ collision mode with optimized parameters: RF power 1550 W (for plasma stability), gas flows (plasma 15.0 L min⁻¹, auxiliary 1.0 L min⁻¹, carrier 0.85 L min⁻¹), sampling depth 5.0 mm, spray chamber temperature 4 °C. Ion lens voltages were calibrated for each element. Time‑resolved analysis with element‑specific dwell times ensured accurate trace quantification and minimized spectral interferences.

### Comprehensive metabolomic profiling of potato tuber flesh

For untargeted metabolomics, 0.1 g tuber flesh was homogenized in 2 mL microcentrifuge tubes containing 75% methanol–chloroform (9:1, v/v), 25% H₂O, and stainless‑steel beads. Samples underwent two 60‑s homogenization cycles (50 Hz), 30 min sonication (25 ± 1 °C), and 30 min cold incubation (0–4 °C). After centrifugation, pellets were vacuum‑dried and reconstituted in the extraction solution described for phenolic acid and flavonoid analysis.

Chromatographic separation^59^ was conducted on a Thermo Vanquish UHPLC system equipped with an ACQUITY UPLC HSS T3 column (2.1 × 100 mm, 1.8 µm) maintained at 40 °C. The flow rate was 0.3 mL/min with a 2 µL injection volume. Positive and negative ionization modes were analyzed in separate runs. In positive mode, mobile phases consisted of 0.1% formic acid in water (A) and 0.1% formic acid in acetonitrile (B), with the following gradient: 0–1 min, 10% B; 1–5 min, 10–98% B; 5–6.5 min, 98% B; 6.5–6.6 min, 98–10% B; 6.6–8 min, 10% B. In negative mode, 5 mM ammonium formate in water (A) and acetonitrile (B) were used with an identical gradient profile. Mass spectrometry^60^ was performed on a Thermo Orbitrap Exploris 120 mass spectrometer with electrospray ionization (ESI). Source parameters were: spray voltages of 3.50 kV (positive) and 2.50 kV (negative), sheath gas 40 arb, auxiliary gas 10 arb, and capillary temperature 325 °C. Full-scan MS data (*m/z* 100–1000) were acquired at 60,000 resolution, with data-dependent MS/MS acquisition selecting the top 4 ions for fragmentation using HCD (30% NCE) at 15,000 resolution, with dynamic exclusion enabled. Raw data files were converted to mzXML format using MSConvert (ProteoWizard, v3.0.8789). Peak detection, alignment, and integration were performed using the XCMS package in R with parameters: bw = 2, ppm = 15, peakwidth = c(5, 30), mzwid = 0.015, mzdiff = 0.01, and method = “centWave”. The resulting feature table was normalized to total ion current (TIC). Putative metabolite identification was achieved by matching accurate mass and MS/MS spectra against the mzCloud and mzVault databases (mass tolerance: 5 ppm). Metabolites with a variable importance in projection (VIP) score >1.0, |log₂(fold change)| ≥1, and *p* < 0.05 were considered differentially abundant. Raw LC–MS data are available via MetaboLights (accession MTBLS13298).

### Integrative transcriptome profiling and gene co‑expression network analysis

Total RNA was extracted from mature tuber flesh (*n* = 15 biological replicates) using the Quick RNA Isolation Kit (Huayueyang Biotechnology, China). The concentration, purity, and integrity of the RNA were assessed using a NanoDrop spectrophotometer and an Agilent Bioanalyzer. Sequencing libraries were prepared and sequenced (150 bp paired‑end) on an Illumina NovaSeq6000 platform. Raw reads were subjected to quality control using FastQC, and adapters and low-quality bases were trimmed using Trimmomatic. Clean reads were aligned to the potato reference genome (DMv6.1) using HISAT2 (v2.2.1). Gene-level read counts were generated using featureCounts^47^ with the parameter -pto count fragments.

Differential expression analysis was performed with DESeq2 in R; genes with an adjusted *P*-value (FDR) < 0.05 and |log₂(fold change)| ≥1 were designated as DEGs. Functional enrichment analysis of DEGs was performed based on the Kyoto Encyclopedia of Genes and Genomes (KEGG) and Gene Ontology (GO) databases. Raw sequence data have been deposited in the NCBI Sequence Read Archive (SRA) under BioProject accession number PRJNA1358977. To identify gene modules associated with carotenoid accumulation, WGCNA was performed on all expressed genes using the R package WGCNA. The correlation between module eigengenes and total carotenoid content was calculated to identify carotenoid-associated modules. For integration with metabolomic data, both gene expression (TPM) and metabolite abundance matrices were z-score normalized. Pairwise Pearson correlation coefficients were computed between the expression levels of hub genes from the key carotenoid-associated WGCNA module and the abundance of all significantly altered metabolites. Regulatory networks were constructed by linking genes and metabolites with significant correlations. The resulting networks were visualized and analyzed using Cytoscape (v3.9.1), with nodes representing genes or metabolites and edges representing significant correlations.

### Standardized qPCR Workflow

Gene expression was quantified by RT-qPCR on a 7500 Fast Real-Time PCR System (Applied Biosystems) using intercalating dye chemistry (Takara), per manufacturer’s protocol. Three biological replicates per condition were analyzed; relative expression was calculated by the *ΔC*_*t*_ method with *Actin* as reference. Primer specificity was verified by NCBI BLAST; sequences are in Supplementary Table 3.

### Potato tuber endophytic bacterial community analysis

Genomic DNA was extracted from homogenized tuber flesh using a Qiagen DNA extraction kit. Integrity was checked (Nanodrop2000, Thermo Fisher) prior to PCR amplification of the 16S rRNA gene V3–V4 region (50 ng μL⁻¹ template) with primers (Supplementary Table 3). Amplicons were sequenced on an Illumina NovaSeq platform.

Raw reads were processed through the DADA2 pipeline in QIIME 2 (v2024.5) for quality filtering, denoising, merging, and chimera removal to generate a table of amplicon sequence variants (ASVs). Taxonomy was assigned using the classify-consensus-blast method against the SILVA 138.1 database. Alpha diversity indices were calculated on rarefied data using the *phyloseq* package in R. Beta diversity was assessed with Bray–Curtis dissimilarity and PERMANOVA. Associations between ASV abundance and metabolite profiles were analyzed using Spearman correlation networks ( | ρ | > 0.6, *P* < 0.05) in R.

### Statistical analysis

Data are presented as mean±SD (*n* = 3 biological replicates). One‑way ANOVA with Tukey’s HSD *post* *hoc* test (*P* < 0.05) assessed group differences, denoted by unique lowercase letters (a, b, c, d, e) where *P* < 0.05.

## Supplementary information

41538_2026_842_MOESM1_ESM

41538_2026_842_MOESM2_ESM

41538_2026_842_MOESM3_ESM

41538_2026_842_MOESM4_ESM

41538_2026_842_MOESM5_ESM

41538_2026_842_MOESM6_ESM

41538_2026_842_MOESM7_ESM

41538_2026_842_MOESM8_ESM

41538_2026_842_MOESM9_ESM

41538_2026_842_MOESM10_ESM

41538_2026_842_MOESM11_ESM

## Data Availability

The authors declare that all pertinent data that support this study have been included within the paper. Raw data will be made available by corresponding authors upon request.
