# Supplementary material for: Multi-omics profiling of high-carotenoid hybrid potato lines reveals coordinated metabolic reprogramming and associates with distinct tuber microbiota
Source: NPJ Sci Food. 2026 Jul 4;10:213. doi: 10.1038/s41538-026-00842-3 (PMC13338389; doi:10.1038/s41538-026-00842-3)
Supplement: Supplementary file 11 — 41538_2026_842_MOESM11_ESM [file 41538_2026_842_MOESM11_ESM.pdf]

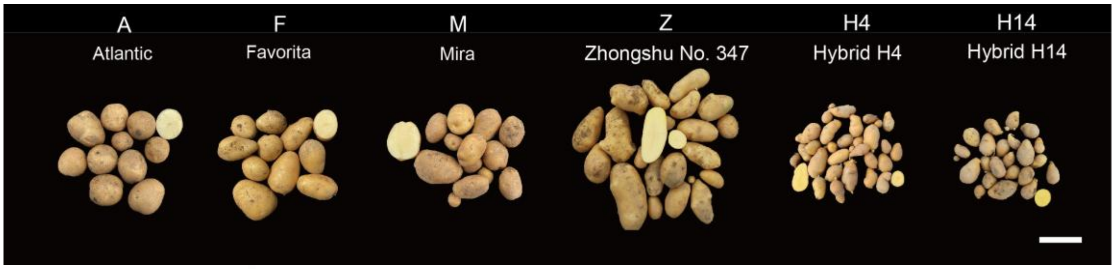

Supplementary Figure 1. Phenotypic characterization of six potato materials. Bar number = 10 cm.

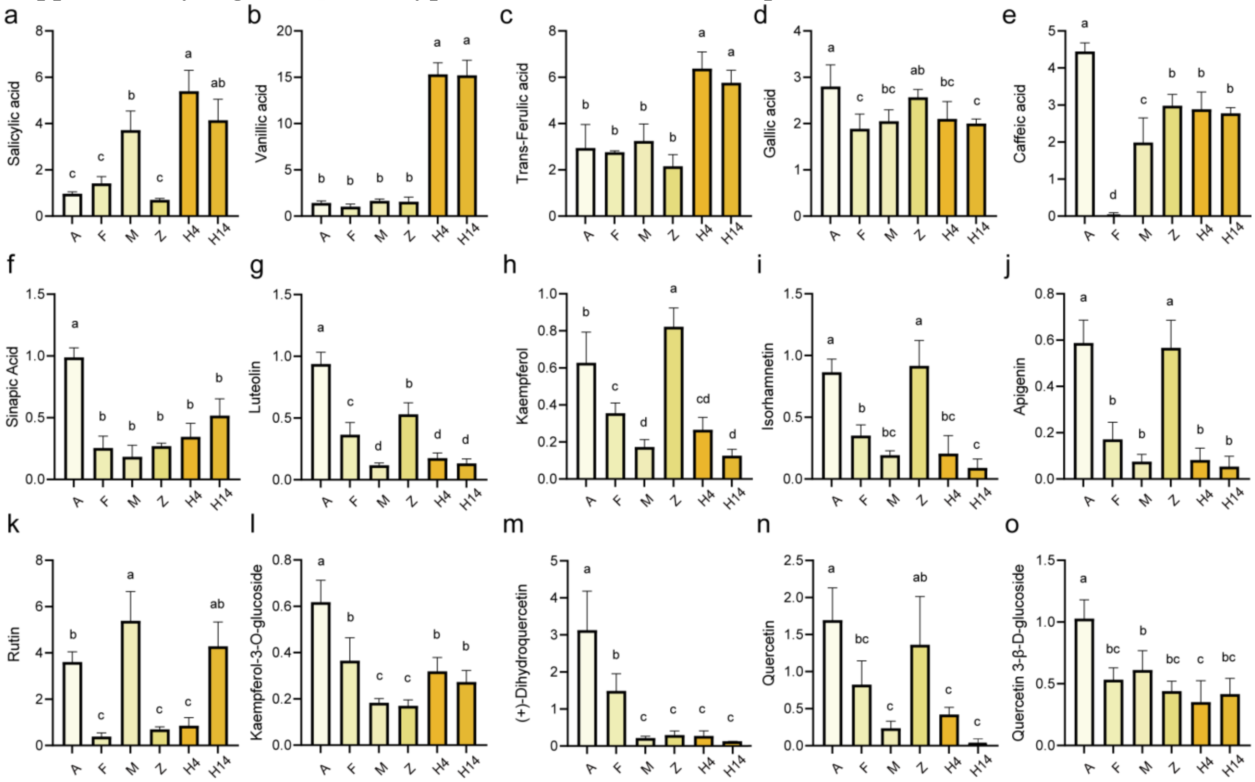

Supplementary Figure 2. Targeted profiling of phenolic acids and flavonoids in six potato tuber flesh samples.a–f Phenolic acid content: salicylic acid (a), vanillic acid (b), trans-ferulic acid (c), gallic acid (d), caffeic acid (d), sinapic acid (f). g–o Flavonoid content: luteolin (g), kaempferol (h), isorhamnetin (i), apigenin (j), rutin (k), kaempferol-3-O-glucoside (l), (+)-dihydroquercetin (m), quercetin (n), quercetin-3-O-glucoside (o). Values are means ± SD ($mg \cdot 100\ g^{-1}$ FW). Bar colors correspond to flesh color of each sample. Different lowercase letters denote significant differences among samples ($P < 0.05$)

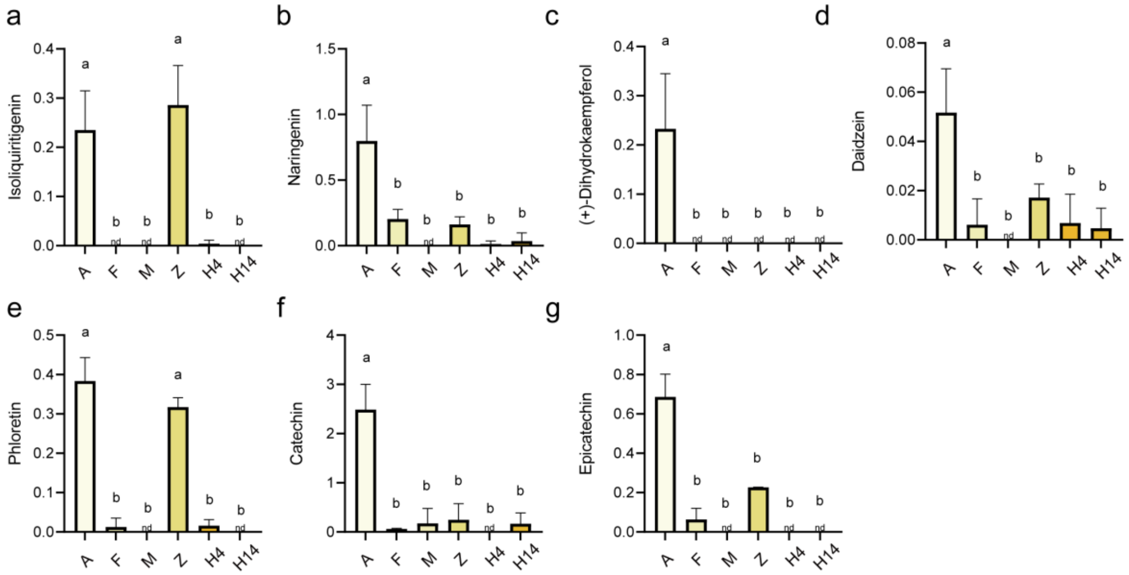

Supplementary Figure 3. Flavonoid content in six potato samples. (nd: Not Detected in some samples due to levels below the detection limit)

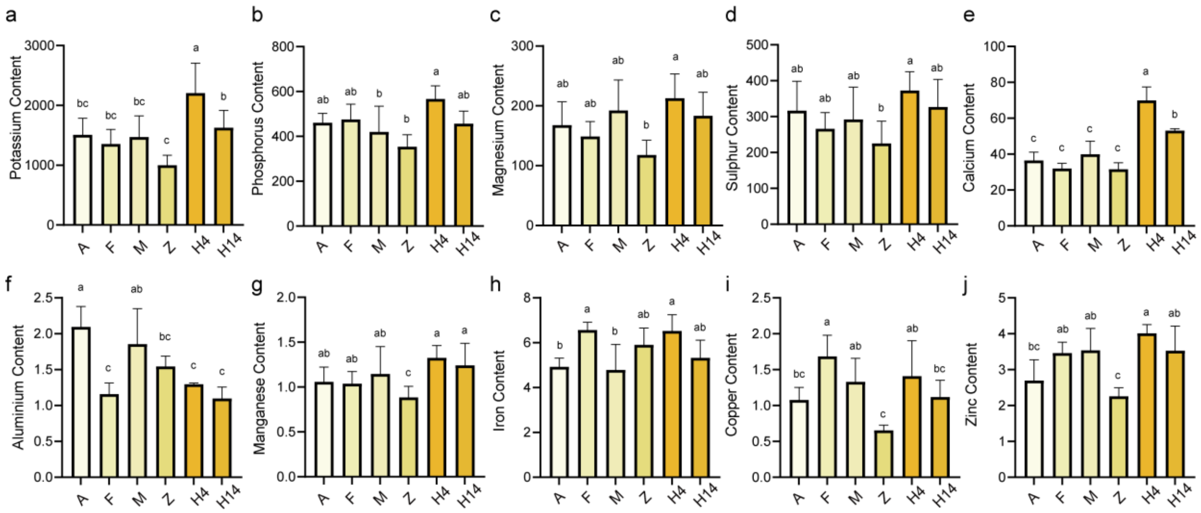

Supplementary Figure 4. Macronutrient and micronutrient composition in tuber flesh of six potato samples. a–e Macronutrient content: potassium (a), phosphorus (b), magnesium (c), sulfur (d), calcium (e). f–j Micronutrient content: aluminum (f), manganese (g), iron (h), copper (i), zinc (j). Values are means ± SD ($mg \cdot kg^{-1}$ FW). Bar colors correspond to flesh color of each sample. Different lowercase letters denote significant differences among samples ($P < 0.05$).

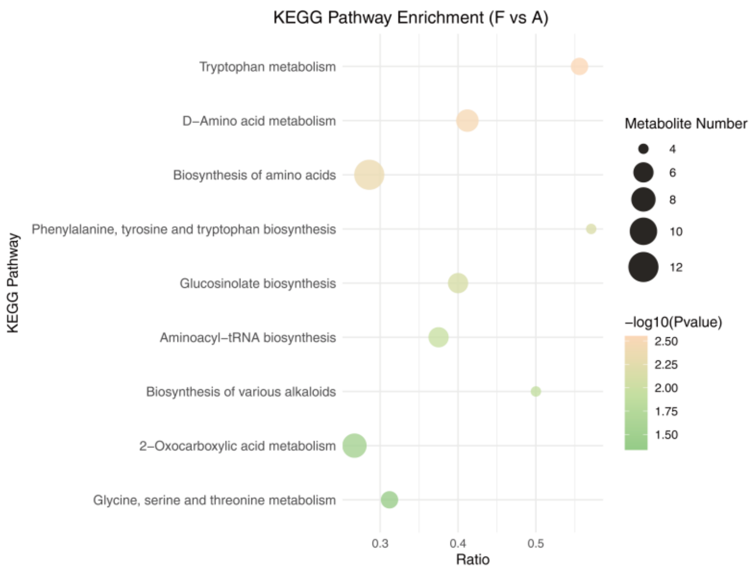
KEGG Pathway Enrichment (F vs A)
Tryptophan metabolism
D−Amino acid metabolism
Biosynthesis of amino acids
Phenylalanine, tyrosine and tryptophan biosynthesis
Glucosinolate biosynthesis
Aminoacyl−tRNA biosynthesis
Biosynthesis of various alkaloids
2−Oxocarboxylic acid metabolism
Glycine, serine and threonine metabolism
KEGG Pathway
0.3
0.4
0.5
Ratio
Metabolite Number
4
6
8
10
12
−log10(Pvalue)
2.50
2.25
2.00
1.75
1.50

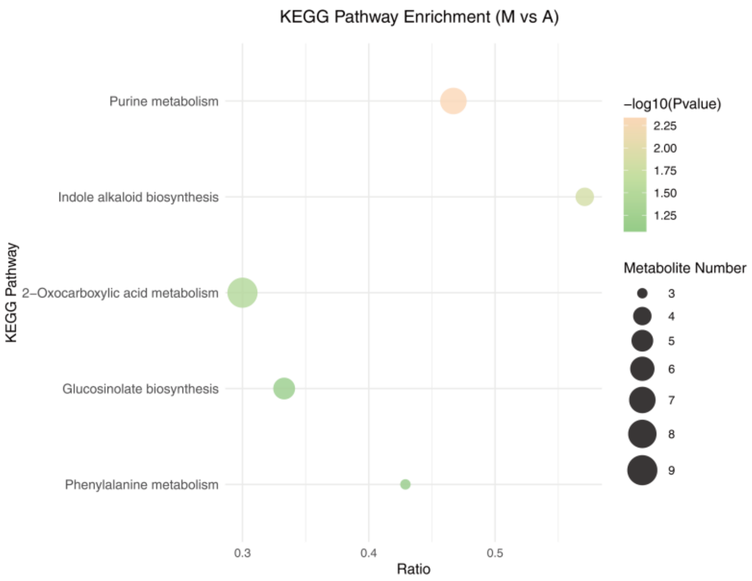
KEGG Pathway Enrichment (M vs A)
Purine metabolism
Indole alkaloid biosynthesis
2−Oxocarboxylic acid metabolism
Glucosinolate biosynthesis
Phenylalanine metabolism
KEGG Pathway
0.3
0.4
0.5
Ratio
−log10(Pvalue)
2.25
2.00
1.75
1.50
1.25
Metabolite Number
3
4
5
6
7
8
9

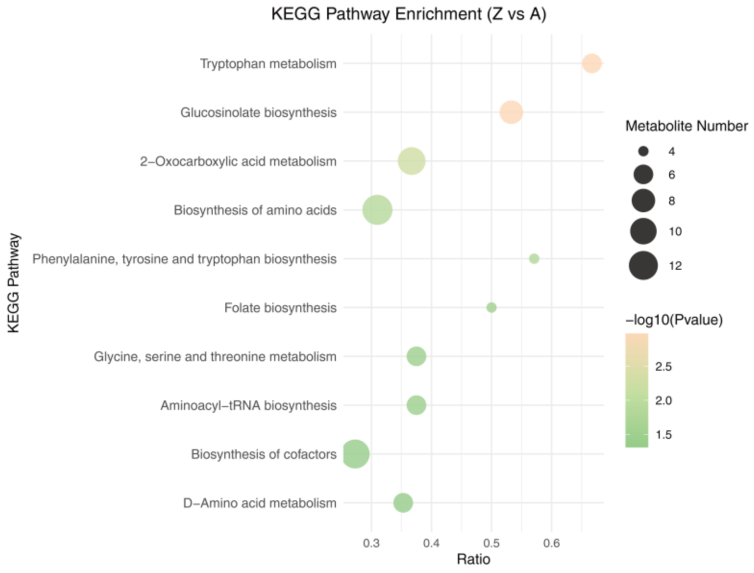
KEGG Pathway Enrichment (Z vs A)
Tryptophan metabolism
Glucosinolate biosynthesis
2−Oxocarboxylic acid metabolism
Biosynthesis of amino acids
Phenylalanine, tyrosine and tryptophan biosynthesis
Folate biosynthesis
Glycine, serine and threonine metabolism
Aminoacyl−tRNA biosynthesis
Biosynthesis of cofactors
D−Amino acid metabolism
KEGG Pathway
0.3
0.4
0.5
0.6
Ratio
Metabolite Number
4
6
8
10
12
−log10(Pvalue)
2.5
2.0
1.5

Supplementary Figure 5. KEGG pathway enrichment of differentially abundant metabolites (F, M, Z vs. A)

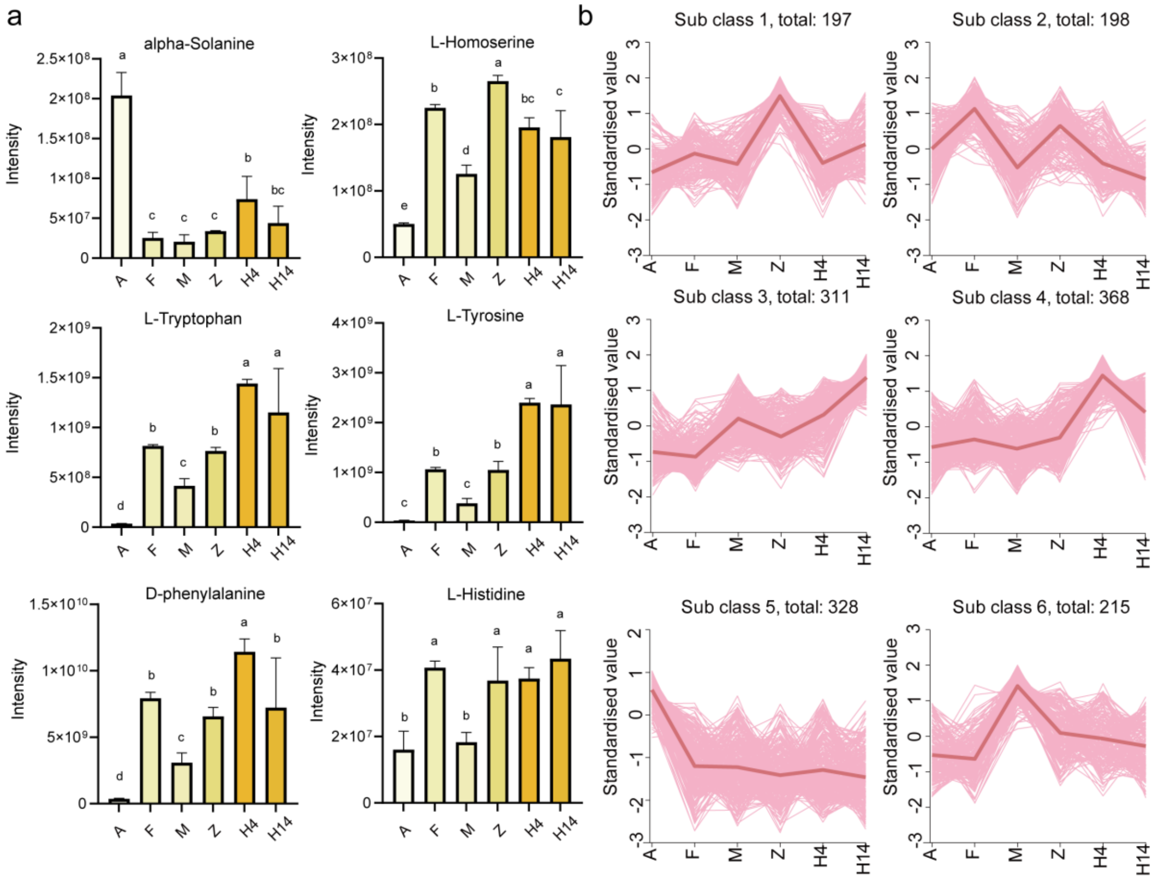

Supplementary Figure 6. **a** Variations in metabolite peaks showing significant differences between Group A and the other treatment groups (F, M, Z, H4, H14).**b** K-means clustering analysis of metabolite peaks based on their abundance or intensity profiles.

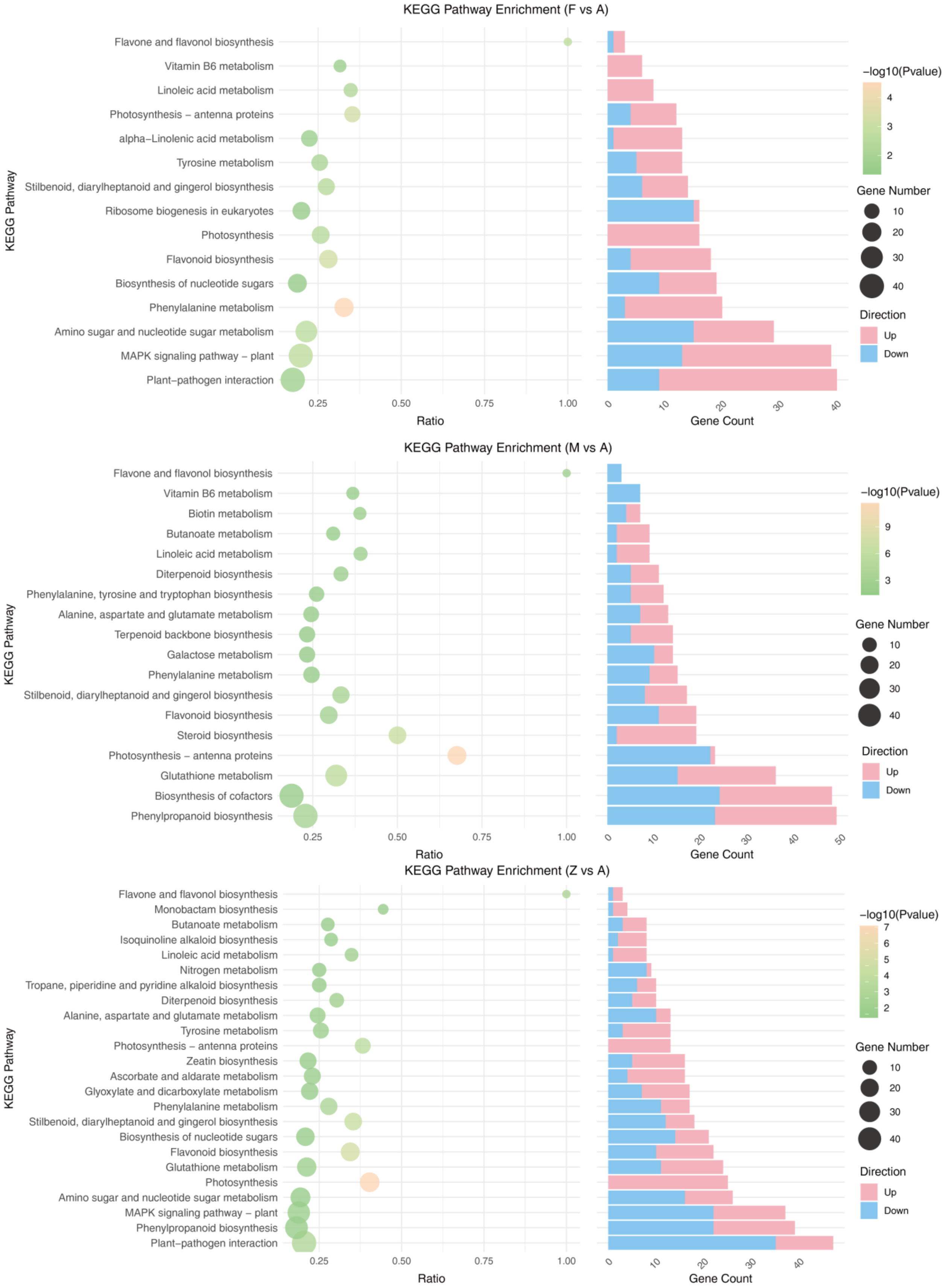
KEGG Pathway Enrichment (F vs A)
KEGG Pathway
Flavone and flavonol biosynthesis
Vitamin B6 metabolism
Linoleic acid metabolism
Photosynthesis – antenna proteins
alpha–Linolenic acid metabolism
Tyrosine metabolism
Stilbenoid, diarylheptanoid and gingerol biosynthesis
Ribosome biogenesis in eukaryotes
Photosynthesis
Flavonoid biosynthesis
Biosynthesis of nucleotide sugars
Phenylalanine metabolism
Amino sugar and nucleotide sugar metabolism
MAPK signaling pathway – plant
Plant–pathogen interaction
0.25
0.50
0.75
1.00
Ratio
0
10
20
30
40
Gene Count
–log10(Pvalue)
4
3
2
Gene Number
10
20
30
40
Direction
Up
Down
KEGG Pathway Enrichment (M vs A)
KEGG Pathway
Flavone and flavonol biosynthesis
Vitamin B6 metabolism
Biotin metabolism
Butanoate metabolism
Linoleic acid metabolism
Diterpenoid biosynthesis
Phenylalanine, tyrosine and tryptophan biosynthesis
Alanine, aspartate and glutamate metabolism
Terpenoid backbone biosynthesis
Galactose metabolism
Phenylalanine metabolism
Stilbenoid, diarylheptanoid and gingerol biosynthesis
Flavonoid biosynthesis
Steroid biosynthesis
Photosynthesis – antenna proteins
Glutathione metabolism
Biosynthesis of cofactors
Phenylpropanoid biosynthesis
0.25
0.50
0.75
1.00
Ratio
0
10
20
30
40
50
Gene Count
–log10(Pvalue)
9
6
3
Gene Number
10
20
30
40
Direction
Up
Down
KEGG Pathway Enrichment (Z vs A)
KEGG Pathway
Flavone and flavonol biosynthesis
Monobactam biosynthesis
Butanoate metabolism
Isoquinoline alkaloid biosynthesis
Linoleic acid metabolism
Nitrogen metabolism
Tropane, piperidine and pyridine alkaloid biosynthesis
Diterpenoid biosynthesis
Alanine, aspartate and glutamate metabolism
Tyrosine metabolism
Photosynthesis – antenna proteins
Zeatin biosynthesis
Ascorbate and aldarate metabolism
Glyoxylate and dicarboxylate metabolism
Phenylalanine metabolism
Stilbenoid, diarylheptanoid and gingerol biosynthesis
Biosynthesis of nucleotide sugars
Flavonoid biosynthesis
Glutathione metabolism
Photosynthesis
Amino sugar and nucleotide sugar metabolism
MAPK signaling pathway – plant
Phenylpropanoid biosynthesis
Plant–pathogen interaction
0.25
0.50
0.75
1.00
Ratio
0
10
20
30
40
Gene Count
–log10(Pvalue)
7
6
5
4
3
2
Gene Number
10
20
30
40
Direction
Up
Down

Supplementary Figure 7. KEGG pathway enrichment analysis of differentially expressed genes in comparisons of F, M, Z vs. A

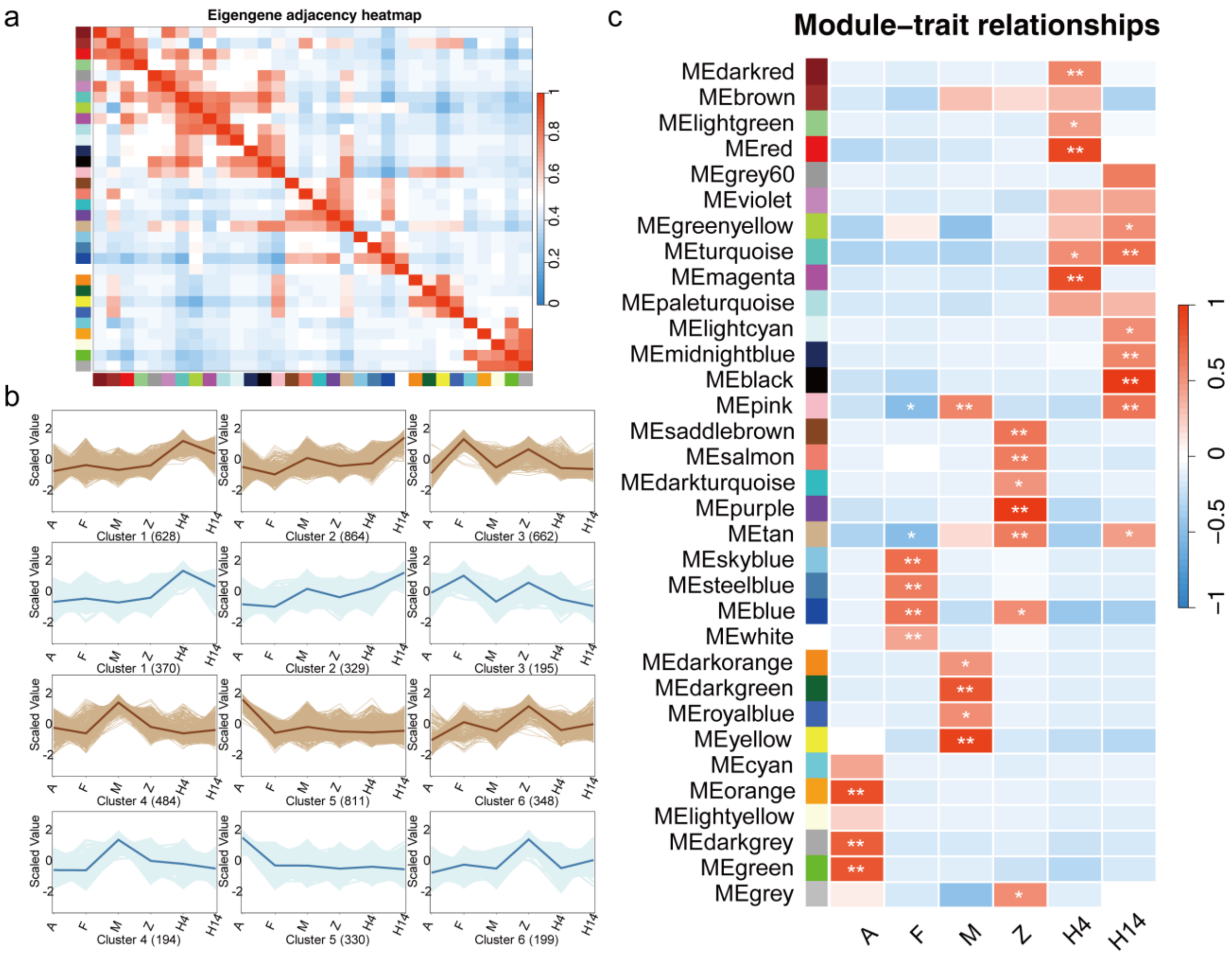

Supplementary Figure 8. Weighted gene co-expression network analysis (WGCNA) and multi-omics integration reveal coordinated transcriptional and metabolic modules. **a** Eigengene adjacency heatmap illustrating the pairwise correlation relationships among the 33 gene modules identified by WGCNA. **b** Integrated k-means clustering of transcriptomic (brown) and metabolomic (blue) profiles, identifying six distinct gene–metabolite co-clusters. **c** Heatmap of module–trait correlations, depicting the association between each gene module eigengene and key phenotypic traits across the six potato genotypes. Statistical significance is indicated by asterisks: $P < 0.05$ * and $P < 0.01$**.

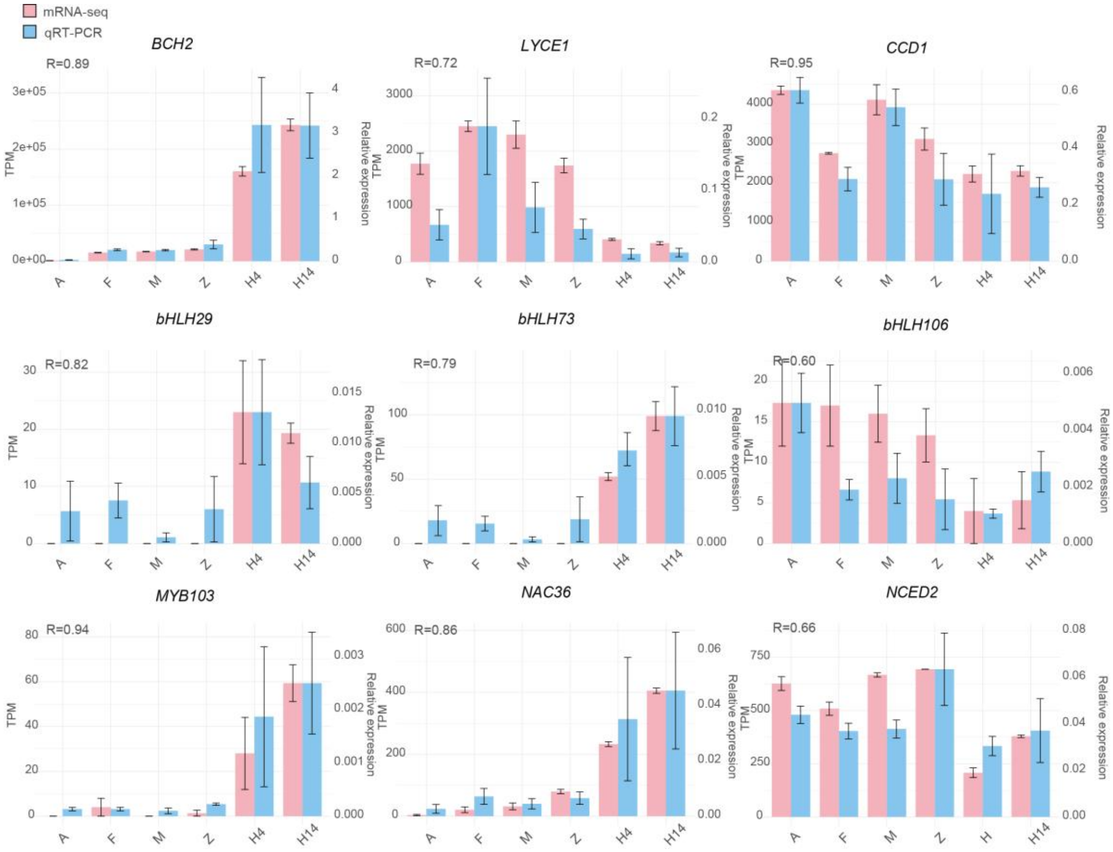

Supplementary Figure 9. Verification for 9 DEGs in potato tubers by qRT-PCR. Error bars represent the standard deviation of three replicates. Pearson's correlation coefficients were calculated by comparing qRT-PCR and RNA-seq data for each gene in all samples.

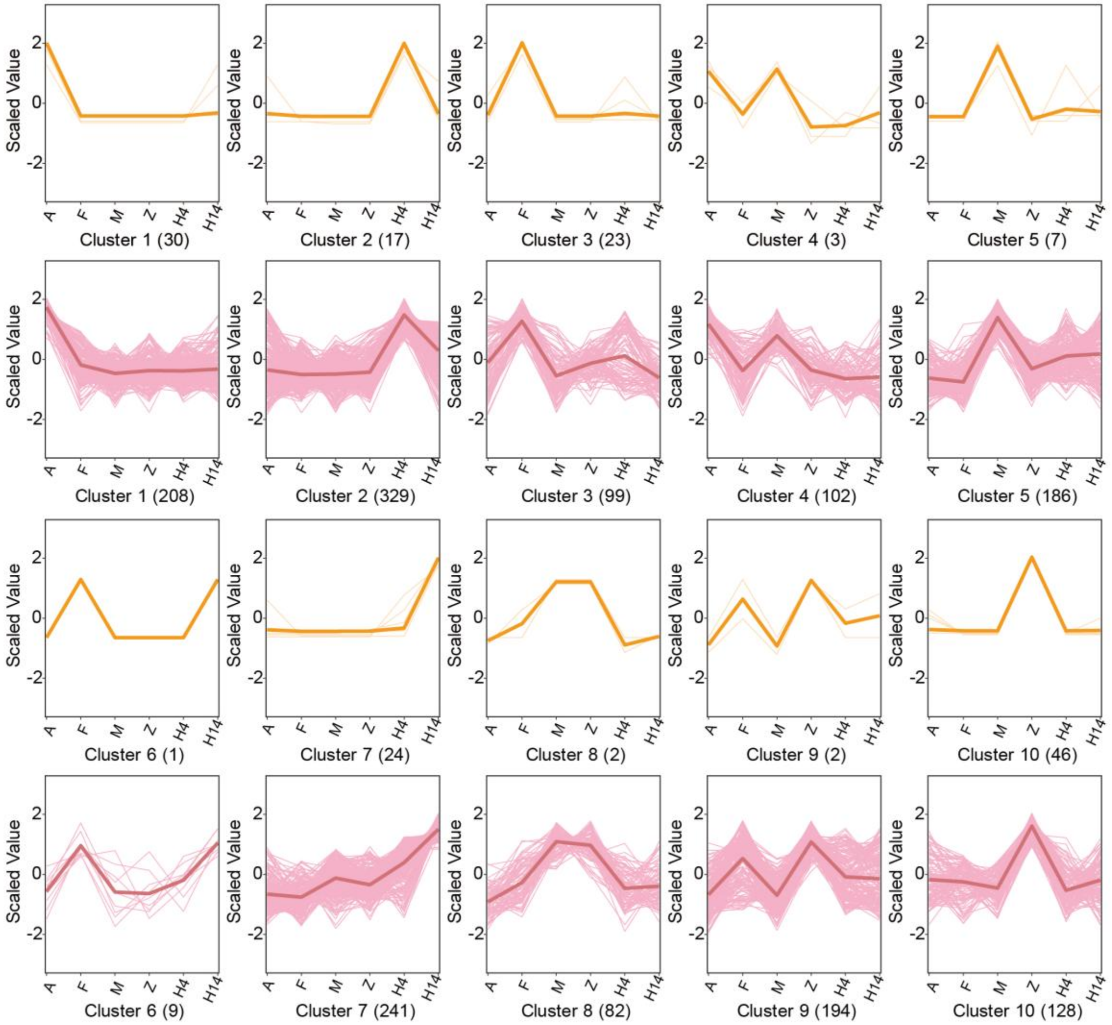

Supplementary Figure 10. K-means clustering graph of ASVs (orange) and metabolites (pink).

Supplementary Table 1. Number of servings (250g fresh weight) of potato varieties needed to meet the Recommended Dietary Allowance (RDA) for different age categories.

Essential Elements	RDA (mg d-1)	Age Group	Potato Varieties					
			Atlantic (A)	Favorita (F)	Mira (M)	Zhongshu No.347 (Z)	Hybrid H4 (H4)	Hybrid H14 (H14)
Copper (Cu)	0.44	Child	1.2±0.1	1.0±0.1	1.1±0.1	1.2±0.1	1.0±0.1	0.9±0.1
	0.9	Adult	2.4±0.2	2.0±0.2	2.2±0.2	2.5±0.2	1.9±0.2	2.0±0.2
	0.9	Elderly	2.4±0.2	2.0±0.2	2.2±0.2	2.5±0.2	1.9±0.2	2.0±0.2
Iron (Fe)	10	Child	4.1±0.4	4.5±0.4	4.9±0.5	5.6±0.6	3.6±0.4	4.1±0.4
	13	Adult	5.3±0.5	5.8±0.6	6.4±0.6	7.3±0.7	4.7±0.5	5.4±0.5
	8	Elderly	3.3±0.3	3.6±0.4	3.9±0.4	4.5±0.4	2.9±0.3	3.3±0.3
Magnesium (Mg)	130	Child	6.1±0.6	6.1±0.6	6.7±0.7	8.2±0.8	6.3±0.6	7.0±0.7
	355	Adult	16.7±1.7	16.6±1.7	18.3±1.8	22.5±2.2	17.1±1.7	19.0±1.9

	370	Elderly	17.5±1.7	17.3±1.7	19.1±1.9	23.4±2.3	17.9±1.8	19.8±2.0
Manganese (Mn)	1.5	Child	2.7±0.3	2.0±0.2	1.6±0.2	1.9±0.2	1.3±0.1	1.4±0.1
	2.05	Adult	3.7±0.4	2.7±0.3	2.2±0.2	2.6±0.3	1.8±0.2	1.9±0.2
	2.05	Elderly	3.7±0.4	2.7±0.3	2.2±0.2	2.6±0.3	1.8±0.2	1.9±0.2
Phosphorus (P)	500	Child	5.6±0.6	6.4±0.6	5.5±0.6	6.3±0.6	5.0±0.5	4.8±0.0
	700	Adult	7.8±0.8	9.0±0.9	7.7±0.8	8.9±0.9	7.0±0.7	6.7±0.7
	700	Elderly	7.8±0.8	9.0±0.9	7.7±0.8	8.9±0.9	7.0±0.7	6.7±0.7
Potassium (K)	2300	Child	21.8±2.2	23.9±2.4	23.2±2.3	26.3±2.6	20.3±2.0	21.9±2.2
	3000	Adult	28.4±2.8	31.2±3.1	30.2±3.0	34.4±3.4	26.5±2.6	28.6±2.9
	3000	Elderly	28.4±2.8	31.2±3.1	30.2±3.0	34.4±3.4	26.5±2.6	28.6±2.9
Zinc (Zn)	5	Child	6.6±0.7	7.7±0.8	5.7±0.6	9.4±0.9	4.5±0.4	4.7±0.5
	9.5	Adult	12.5±1.2	14.6±1.5	10.8±1.1	17.9±1.8	8.5±0.8	9.0±0.9
	9.5	Elderly	12.5±1.2	14.6±1.5	10.8±1.1	17.9±1.8	8.5±0.8	9.0±0.9

Note: Values are presented as mean ± SD. RDA values are based on established dietary guidelines. The formula used: Number of servings = RDA ÷ Mineral content per 250g serving (fresh weight). (RDA data are from the website: https://lpi.oregonstate.edu/mic/minerals)

Supplementary Table 2. Statistical Analysis of 16S rRNA Gene Sequencing Data (V3-V4 Region) for Sample Processing Results.

Sample ID	Raw Reads	Clean Reads	Denoised Reads	Merged Reads	Non-chimeric Reads
A_1	79,770	71,296	71,294	71,247	70,793
A_2	79,960	72,661	72,653	72,226	71,660
A_3	79,974	72,113	72,107	72,098	71,302
F_1	80,101	71,913	71,913	71,853	71,593
F_2	79,829	71,504	71,494	71,444	71,216
F_3	80,191	71,951	71,950	71,519	71,158
M_1	80,007	72,466	72,458	72,422	71,457
M_2	80,106	72,148	72,139	71,975	70,910
M_3	79,965	72,705	72,703	72,200	71,530
Z_1	80,122	72,479	72,468	72,443	72,001
Z_2	80,012	72,194	72,194	72,153	71,594
Z_3	80,068	72,197	72,197	71,987	71,537
H4_1	79,960	71,882	71,880	71,830	70,948
H4_2	79,789	71,579	71,574	71,537	70,716
H4_3	79,831	71,691	71,691	71,627	70,665
H14_1	79,791	71,946	71,943	71,923	71,136
H14_2	80,038	72,344	72,339	72,296	71,659

H14_3	79,975	71,794	71,790	71,772	71,324

Supplementary Table 3. The sequences of the gene-specific primers for study.

Name	primers (5′-3′)	Notes
16S rRNA gene's V3-V4	F:ACTCCTACGGGAGGCAGCA<br>R:GGACTACHVGGGTWTCTAAT	16S rRNA gene's V3-V4
MYB103	F:GTCCCTGAAAAAGCTGGGCT<br>R:CACCTGTTGCCTACAGCTCC	Soltu.DM.08G028250
bHLH106	F:AAGGCCTCTCTTTGTTGCGA<br>R:ACGAATTCTCCCTCCTAACGTG	Soltu.DM.10G029720
bHLH73	F:GGTGGAGGAAACGGTGAACT<br>R:CTTCCACTCTGCTCCGTCAG	Soltu.DM.03G027480
NAC36	F:AGGCAACTGGTTCTGATCGT<br>R:AGGAAGTCTGTACTCATTCATGACC	Soltu.DM.02G005400
bHLH29	F:TCATGCCGCCATTGACAGAT<br>R:ACAACTCAGCCATTTTCCCTCT	Soltu.DM.01G022230
LYCE1	F:CGGTGGTTCGCAACTTGTTT<br>R:CAGCAGGACCACAGCCAATA	Soltu.DM.12G025930
BCH2	F:CATAAAGGCCTCGTCCCTGG<br>R:CGAAAGTAAGGCACGTTGGC	Soltu.DM.03G018410
CCD1	F:TGTTGATTACCTGCCGCCTT<br>R:TGTGAAGCTGCACCACTCTT	Soltu.DM.01G027390
NCED2	F:TCGGGCTTGTGGATCATAGC<br>R:TGAAGATCGCCAGAAGGCAA	Soltu.DM.08G006990
Actin	F:GGATCTTGCTGGTCGTGATTTAAC<br>R:CATAGGCAAGCTTTTCCTTCATGT	Soltu.DM.11G008990

Supplementary Data 1. Metabolites detected by non-targeted metabolomics.
Supplementary Data 2. Differentially accumulated genes between H4 and A.
Supplementary Data 3. Differentially accumulated genes between H14 and A.
Supplementary Data 4. Differentially accumulated genes between F and A.
Supplementary Data 5. Differentially accumulated genes between M and A.
Supplementary Data 6. Differentially accumulated genes between Z and A.
Supplementary Data 7. Genes list of 33 modules.
Supplementary Data 8. Transcription expression of genes involved in carotenoid biosynthesis.
Supplementary Data 9. The species annotation results of Cluster 2 and Cluster 7.
Supplementary Data 10. The Spearman correlation analysis results between metabolites and ASVs.
